# New distribution records for Canadian Aleocharinae (Coleoptera, Staphylinidae), and new synonymies for *Trichiusa*

**DOI:** 10.3897/zookeys.498.9282

**Published:** 2015-04-21

**Authors:** Jan Klimaszewski, Benoit Godin, David Langor, Caroline Bourdon, Seung-Il Lee, Denise Horwood

**Affiliations:** 1 Natural Resources Canada, Canadian Forest Service, Laurentian Forestry Centre, 1055 du P.E.P.S., P.O. Box 10380, Stn. Sainte-Foy, Québec, Quebec, Canada G1V 4C7; 2 14A Thompson, Whitehorse, Yukon, Canada Y1A 0C4; 3 Natural Resources Canada, Canadian Forest Service, Northern Forestry Centre, 5320-122 Street, 751 General Services Building, Edmonton, Alberta, Canada T6H 3S5; 4 Department of Renewable Resources, University of Alberta, 230E Earth Sciences Building, Edmonton, Alberta, Canada T6G 2H1

**Keywords:** Coleoptera, rove beetles, Staphylinidae, new distribution records, new synonymy, species richness, Canada, Alberta, British Columbia, Manitoba, Northwest Territories, Saskatchewan, Yukon Territory

## Abstract

Fifty-four new Canadian provincial records of aleocharine beetles (Staphylinidae), including three new Canadian records and one new North American record, are presented. Of these, 33 are new provincial records for Saskatchewan, 14 for Alberta, two for British Columbia, three for Manitoba, two for the Northwest Territories and one for the Yukon Territory. The following are new Canadian records: *Trichiusa pilosa* Casey [formerly reported from Nova Scotia and Ontario as *Trichiusa postica* Casey], *Acrotona recondita* (Erichson) and the adventive Palaearctic *Atheta nigra* (Kraatz), which is also a new North American record. Bionomics information and new locality records are provided. The following new synonyms of *Trichiusa pilosa* Casey are established: *Trichiusa atra* Casey, *Trichiusa monticola* Casey, *Trichiusa parviceps* Casey, and *Trichiusa postica* Casey. The numbers of Aleocharinae remaining to be discovered in Canadian provinces and territories are discussed.

## Introduction

Staphylinidae (the rove beetles) are the most species-rich family of beetles in Canada with 1652 species and subspecies recorded, 510 of which are in the Aleocharinae (Bousquet et al. 2013). Aleocharinae are one of the poorest known subfamilies of rove beetles in Canada, although enormous strides have been made in understanding the taxonomy of this group in the last 20 years, especially in eastern Canada. Western and northern Canada (Manitoba to British Columbia, and the three territories), however, remain poorly studied except for a few localities in coastal British Columbia (Klimaszewski and Winchester 2002, McLean et al. 2009a, b) and in the Yukon (Klimaszewski et al. 2008, 2012). Thus the full distribution of many species recorded for Canada is not known because of the large gaps in sampling intensity. Nonetheless, the fauna of these provincial and territorial jurisdictions is starting to receive more attention as many studies in recent years have sampled aleocharines in a large number of habitats, particularly in Alberta. Improved sampling of Staphylinidae and especially Aleocharinae are needed to establish baseline biodiversity composition in areas of the country where ecosystems are undergoing rapid change due to resource extraction and climate change. As well, this family and subfamily are known to be exceptionally good ecological indicators and are increasingly being used to assess ecosystem resistance and resilience in the wake of development and environmental changes (Pohl et al. 2007, 2008, Langor, unpublished data). This paper contributes to improved baseline knowledge of the Aleocharinae in the Canadian west and north by providing 54 new provincial and territorial records for 51 species.

## Materials and methods

All specimens in this study were dissected to examine the genital structures. Extracted genital structures were dehydrated in absolute alcohol, mounted in Canada balsam on celluloid micro-slides, and pinned with the specimens from which they originated. Images of the entire body and the genital structures were taken using an image processing system (Nikon SMZ 1500 stereoscopic microscope; Nikon Digital Camera DXM 1200F, and Adobe Photoshop software).

Morphological terminology mainly follows that used by Seevers (1978) and Klimaszewski et al. (2011). The ventral side of the median lobe of the aedeagus is considered to be the side of the bulbus containing the foramen mediale, the entrance of the ductus ejaculatorius, and the adjacent ventral side of the tubus of the median lobe with the internal sac and its structures (this part is referred to as the parameral side in some recent publications); the opposite side is referred to as the dorsal part. In the species descriptions, microsculpture refers to the surface of the upper forebody (head, pronotum and elytra).

## Depository/institutional abbreviations

BGC Benoit Godin Collection, Whitehorse, Yukon, Canada.

LFC Natural Resources Canada, Canadian Forest Service, Laurentian Forestry Centre, R. Martineau Insectarium, Quebec City, Quebec, Canada.

NoFC Natural Resources Canada, Canadian Forest Service, Northern Forestry Centre, Arthropod Museum, Edmonton, Alberta, Canada.

**Abbreviations of Canadian provinces and territories**

AB – Alberta;

BC – British Columbia;

LB – Labrador;

MB – Manitoba;

NB – New Brunswick;

NF – Newfoundland;

NS – Nova Scotia;

NT – Northwest Territories;

NU – Nunavut;

ON – Ontario;

PE – Prince Edward Island;

QC – Quebec;

SK – Saskatchewan;

YT – Yukon Territory.

USA state abbreviations follow those of the US Postal Service.

## Discussion

Our knowledge of the diversity and distribution of Aleocharinae in Canada has increased rapidly over the last ~25 years, faster than for other subfamilies of rove beetles and for most (perhaps all) families of beetles. This increase in knowledge is attributed to a surge in sampling of this subfamily, particularly in eastern Canada, and to a large amount of taxonomic activity (e.g., by Gusarov, Lohse, Klimaszewski, Webster). Of the 463 native species of Aleocharinae recorded in Canada by Bousquet et al. (2013), 32.8% (152 species) were described in the last 25 years. Many other previously described native species were first recorded in Canada over the last 25 years. Of the 47 non-native species in Canada, 10 (21.3%) were detected in the last 25 years (Klimaszewski et al. 2001, 2002, 2006, 2007a, 2010, 2011, Majka and Klimaszewski 2010, McLean et al. 2009a). Furthermore, recent reviews or revisions have resulted in species keys for no less than 35 genera with Canadian representatives.

Despite the great improvements in taxonomic knowledge and availability of diagnostic resources (keys, illustrations, expertly identified reference material), sampling of most of the microhabitats and regions of the country is still woefully incomplete. In recent years, thanks to very active sampling in the Atlantic provinces (especially New Brunswick and Newfoundland and Labrador) and in the Yukon Territory, coupled with large efforts to identify material and publish findings (Klimaszewski et al. 2005, 2007b, 2008, 2009a, b, 2010, 2011, 2012, Majka and Klimaszewski 2010, Webster et al. 2009, 2012), those are undoubtedly the best-studied regions of the country in terms of the aleocharine fauna. Some small areas of Quebec, Ontario, and coastal British Columbia have also received intensive sampling coupled with expert identification of material in recent years (Klimaszewski and Winchester 2002, Brunke et al. 2012). However, the large majority of central, western and northern Canada remains poorly studied. Large numbers of aleocharines (and other staphylinids) have been collected over the last 25 years as a result of numerous trapping studies in forests, native grasslands, agricultural lands, and wetlands, especially in Alberta. This has resulted in the collection of an estimated 50,000–70,000 specimens of Aleocharinae, especially in epigaeic and saproxylic habitats of forests, throughout much of the province. The vast majority of these specimens have not been prepared or identified. The identification of a few specimens from one small study in northwestern Alberta resulted in the 14 new provincial records reported herein. Similarly, dozens of other sites have been subjected to intensive insect trapping, especially in British Columbia, Quebec and northern Canada, resulting in collection of tens of thousands of aleocharine specimens, the vast majority of which remain undetermined. Virtually every insect collection in the country, and many in other countries, have hundreds to many thousands of undetermined aleocharine specimens. Thus, even modest efforts at determination are sure to reveal new provincial, territorial, national and North American records, and undescribed species. Thus it is not surprising nor unusual that examination of a relatively small number of specimens from a few sites in Saskatchewan resulted in 33 new provincial records, including two new Canadian records and one new North American record. This sampling effort more than doubled the previously known species for the province, now numbering 53 species (Table 1).

**Table 1.** Species of Aleocharinae recorded from SK and their provincial and territorial distribution within Canada and new records of species from AB, BC, MB and YT. Provinces and territories in bold denote new records given in the present publication. Species marked with (†) indicate adventive species and species marked with (*) are Holarctic.

**Tribe ALEOCHARINI**	
*Aleochara assiniboin* Klimaszewski	BC, MB, ON, SK, YT
*Aleochara bilineata* Gyllenhal†	AB, BC, MB, NB, NF, NS, ON, PE, QC,SK
*Aleochara bimaculata* Gravenhorst	AB, BC, LB, MB, NB, NF, NS, ON, QC, SK, NT
*Aleochara gracilicornis* Bernhauer	BC, MB, NB, NS, NT, ON, QC, SK
*Aleochara lacertina* Sharp	AB, BC, MB, NB, NF, NS, ON, QC, SK
*Aleochara lata* Gravenhorst†	BC, MB, ON, QC, SK, YT
*Aleochara sekanai* Klimaszewski	AB, LB, MB, NB, NT, ON, **SK**, YT; USA: AK
*Aleochara tahoensis* Casey	AB, BC, MB, NB, NS, NT, ON, SK, YT
*Aleochara verna* Say	AB, BC, LB, MB, NB, NF, NS, ON, PE, QC, SK, YT; USA: AK
*Tinotus morion* (Gravenhorst) †	AB, BC, NB, NF, NS, ON, QC, **SK**; USA: CT, NV
**Tribe ATHETINI**	
*Acrotona recondita* (Erichson) **new country record**	**SK**; USA: AR, CA, NH, NV, PA
*Aloconota sulcifrons* (Stephens) †	**MB**, NB, NF, ON, QC; USA: AL, IL, IN, KY, MO, NH, NY, TN, VA, WV
*Atheta celata* (Erichson) *	BC, NB, NF, NS, QC, **SK**; USA: AK
*Atheta cryptica* (Lohse, 1990)	**BC**, NF, QC, YT
*Atheta dadopora* C.G. Thomson *	AB, BC, LB, NB, NF, NS, ON, PE, **SK**, YT; USA: AK, NY, PA, RI
*Atheta fanatica* Casey	**AB**, BC, LB, NB, NS, QC, **SK**, YT; USA: AK, NV
*Atheta graminicola* (Gravenhorst) *	AB, BC, LB, MB, NB, NF, NT, ON, QC, **SK**, YT; USA: AK, OR
*Atheta klagesi* Bernhauer	AB, BC, NB, NF, NS, ON, PE, QC, **SK**, YT; USA: IA, ME, MN, NJ, NY, PA
*Atheta longicornis* (Gravenhorst) †	BC, NB, NF, NS, QC, **SK**; USA: CA, MN
*Atheta nigra* (Kraatz) † **new North American record**	**SK**
*Atheta platonoffi* Brundin*	AB, BC, LB, NB, NF, NS, ON, **SK**, YT; USA: AK
*Atheta prudhoensis* (Lohse)	BC, LB, NB, NF, NS, ON, QC, **SK**, YT; USA: AK, VT
*Atheta pseudosubtilis* Klimaszewski & Langor	**AB**, LB, NB, NF, QC
*Atheta ventricosa* Bernhauer	AB, BC, LB, NB, NF, NS, ON, QC, **SK**, YT; USA: AK, DC, NC, NJ, NY, PA, VT
*Boreophilia davidgei* Klimaszewski & Godin	**AB**, YT
*Boreophilia islandica* (Kraatz)*	**AB**, NF, NT, NU, YT; USA: AK; Palaearctic: Europe, Asia (Holarctic species)
*Boreostiba parvipennis* (Bernhauer)	AB, LB, NF, **NT**, QC, YT; USA: AK, NH
*Dinaraea pacei* Klimaszewski & Langor	AB, BC, LB, NB, NF, QC, **YT**; USA: AK
*Dinaraea worki* Klimaszewski & Jacobs	**AB**, QC
*Liogluta aloconoides* Lohse	**AB**, LB, NF, NS, YT
*Lypoglossa franclemonti* Hoebeke	AB, MB, NB, NF, NS, NT, ON, QC, **SK**, YT; USA: NY, VT
*Philhygra botanicarum* (Muona) *	BC, LB, NB, NF, NS, ON, **SK**, YT
*Philhygra clemens* (Casey)	**MB**, NB, NS, ON, QC, YT; USA: WI
*Philhygra jarmilae* Klimaszewski & Langor	NB, NF, ON, **SK**, YT
*Philhygra ripicoloides* Lohse	NF, NT, **SK**, YT
*Philhygra rostrifera* Lohse	LB, **NT**, **SK**, YT; USA: AK
*Philhygra sinuipennis* Klimaszewski & Langor	NB, LB, NF, **SK**, YT
*Philhygra terrestris* Klimaszewski & Godin	**SK**, YT
*Schistoglossa campbelli* Klimaszewski	**AB**, BC
*Schistoglossa carexiana* Klimaszewski	BC, **SK**?
*Schistoglossa hampshirensis* Klimaszewski	**AB**, NB, QC; USA: NH
*Seeversiella globicollis* (Bernhauer)	AB, BC, NB, NF, NS, ON, QC, **SK**; USA: AZ, CO, ID, MN, MT, NH, SD, WI; Mexico; Guatemala
*Trichiusa pilosa* Casey **new country record under this name**	**AB**, **BC**, NS, ON; USA: ID, IN, KS, OH, RI
**Tribe FALAGRINI**	
*Falagria dissecta* Erichson	AB, BC, MB, NB, NS, ON, QC; across USA
**Tribe GYMNUSINI**	
*Gymnusa campbelli* Klimaszewski	MB, NB, NF, NT, ON, QC, **SK**, YT; USA: AK
**Tribe HOMALOTINI**	
*Gyrophaena criddlei* Casey	LB, MB, NB, ON, **SK**, YT
*Gyrophaena insolens* Casey	BC, LB, MB, NB, NF, ON, **SK**; USA: MI
*Gyrophaena uteana* Casey	AB, BC, NB, ON, QC, **SK**; USA: CA, CO, UT
*Homalota plana* (Gyllenhal) †	**AB**, NB, NF, NS; USA: AK; Palaearctic: Europe, Asia
**Tribe LOMECHUSINI**	
*Xenodusa reflexa* (Walker)	AB, BC, MB, NB, NS, QC, ON, SK
**Tribe MYLLAENINI**	
*Myllaena arcana* Casey	AB, LB, NB, NF, NS, ON, QC, **SK**; USA: AL, FL, IA, IL, MA, NH, NJ; Mexico
*Mylaena insomnis* Casey	AB, BC, LB, MB, NB, NF, NS, NT, ON, QC, SK, YT; USA: AK, ID, MA, MN, WI
**Tribe OXYPODINI**	
*Cratarea suturalis* (Mannerheim) †	BC, LB, NB, NS, ON, SK; USA: IL, MA, MO, PA, SC, VA, VT; Palaearctic region
*Devia prospera* (Erichson) *	AB, BC, LB, MB, NB, NT, ON, **SK**, YT; USA: AK, CO, MI, MN, NM, OR, SD, UT, WA, WY; Palaearctic: Europe, Asia
*Gnypeta caerula* (C.R. Sahlberg) *	AB, BC, LB, MB, NB, NF, NS, NT, ON, PE, QC, SK, YT; USA: AK
*Gnypeta carbonaria* (Mannerheim)	AB, MB, NB, NF, NT, ON, QC, SK; USA: AK
*Gnypeta sellmani* Brundin	LB, MB, NF, NT, QC, SK, YT; USA: AK
*Ocyusa canadensis* Lohse	NB, NF, ON, **SK**, YT; USA: AK
*Oxypoda grandipennis* (Casey)	AB, BC, LB, NB, NF, NS, ON, QC, **SK**, YT; USA: AK, NH
*Oxypoda hiemalis* Casey	**AB**, LB, NB, NF, NS, NT, ON, QC; USA: AK
*Oxypoda lacustris* Casey	AB, BC, LB, **MB**, NB, NF, NS, NT, ON, QC, **SK**, YT; USA: AK
*Oxypoda orbicollis* Casey	AB, LB, NB, NS, ON, QC, **SK**, YT; USA: WI
*Oxypoda pseudolacustris* Klimaszewski	AB, NB, NF, NS, ON, QC, **SK**
*Tachyusa obsoleta* Casey	BC, NB, SK
**Tribe PLACUSINI**	
*Placusa incompleta* Sjöberg †	**AB**, BC, NB, NF, NS, ON, QC; USA: WA; Palaearctic: Europe
*Placusa pseudosuecica* Klimaszewski	**AB**, BC, ON, QC
*Placusa tachyporoides* (Waltl)	**AB**, BC, NB, NS, ON, QC; Palaearctic: Europe
**Number of species: 67** (33 new records for SK, 14 for AB, 2 for BC, 3 for MB, 2 for NT, 1 for YT). 51 species representing 54 new provincial records.	7 adventive and 4 Holarctic species

Estimating the expected number of species of aleocharines in Canada is challenging. One way to do this is to extrapolate based on the species richness patterns for the family Carabidae (ground beetles) that is very well known and surveyed throughout most of Canada. This family frequently co-occurs with aleocharines, especially in epigaeic and saproxylic habitats. The jurisdictions where the aleocharine fauna is best known are Yukon Territory, New Brunswick and Newfoundland and Labrador. For Carabidae, the fauna of these three jurisdictions represent 20.5%, 34.3%, and 19.5% of the total number (972) of carabid species/subspecies in Canada (Bousquet et al. 2013). If it is assumed that the known aleocharine fauna of each of these jurisdictions represents a similar percent of the total Canadian fauna, then an extrapolation based on the currently known fauna of Yukon, New Brunswick and Newfoundland and Labrador estimates 630, 601 and 890 species in the Canadian fauna, respectively. It is likely that the actual number falls somewhere in the middle of this range. Thus, it is reasonable to use the average of these three estimates, 707, as the expected species richness for the Canadian fauna, meaning that at least 200 more aleocharine species are expected to be found in Canada. Likely a large proportion of these will be found in British Columbia and southern Ontario and Quebec.

The expected species richness of aleocharines for each jurisdiction can also be estimated using the proportion of the total Canadian carabid fauna in each territory and province and multiplying that against the expected total Canadian aleocharine species richness (707) (Table 2). By comparing this estimated species richness to the actual one (Bousquet et al. 2013), the percent of each jurisdictional fauna documented to date can be calculated. In terms of the percent of fauna documented at the time Bousquet et al. (2013) was published, the most poorly known jurisdictions were Saskatchewan (11%) and Prince Edward Island (19%), followed by Manitoba, Alberta, Northwest Territories, and British Columbia (Table 2). With the 33 new records provided for Saskatchewan in this paper, the percent of expected fauna documented has now risen to 24%. In terms of the expected number of species remaining to be documented, the three prairie provinces and British Columbia each have about 200 species that are yet to be documented, and Ontario and Quebec each have about 150 species to be discovered. Despite the enormous advances in documenting Aleocharinae diversity in Canada over the last 2-3 decades, clearly much remains to be done.

**Table 2.** Number of aleocharine species in Canada and projection of yet undiscovered species per province and territory.

	Provinces and territories													
	YK	NT	NU	BC	AB	SK	MB	ON	QC	NB	NS	PE	NL	Total
Aleocharinae species richness (Bousquet et al. 2013)	129	55	14	175	89	27	73	228	192	206	124	24	174	510
Proportion of total fauna in each jurisdiction based on carabid data (Bousquet et al. 2013)	0.205	0.223	0.035	0.519	0.428	0.359	0.387	0.547	0.493	0.343	0.300	0.179	0.195	
Expected aleocharine species richness extrapolated from carabid diversity data	145	158	25	367	303	254	274	387	348	242	212	126	174	707
Percent of fauna so far documented	89%	35%	56%	48%	29%	11%	27%	59%	55%	85%	58%	19%	<100%	72%
Number of species awaiting discovery	16	103	11	192	214	227	201	159	156	36	88	102	>0	197

### New records

#### ALEOCHARINI Fleming

##### Aleochara (s. str.) sekanai

Taxon classificationAnimaliaColeopteraStaphylinidae

Klimaszewski

Aleochara (s. str.) sekanai (for diagnosis and illustrations, see Klimaszewski et al. 2011)

###### Distribution.

**Table:** Distribution of Aleochara (s. str.) sekanai

Origin	Nearctic
Distribution	Canada: LB, MB, NB, NT, ON, **SK**, YT; USA: AK
New records	New provincial record: **Saskatchewan:** Prince Albert, sandy beach, 53.9804°, -106.28°, 532 m, 4.VI.2013(LFC)1 female
References	Klimaszewski 1984, Gouix and Klimaszewski 2007, Majka and Klimaszewski 2010, Klimaszewski et al. 2011, Bousquet et al. 2013

###### Natural history.

In Saskatchewan, one female was captured on a sandy beach. In Labrador, adults were collected in carrion traps and flight intercept traps in spruce-moss forests (Klimaszewski et al. 2011). Elsewhere, adults were captured from animal carcasses and some from *Carex* and moss near a lake (Klimaszewski 1984). The adults were collected from May to August.

##### Tinotus morion

Taxon classificationAnimaliaColeopteraStaphylinidae

Gravenhorst

Tinotus morion (for diagnosis and illustrations, see Klimaszewski et al. 2011)

###### Distribution.

**Table:** Distribution of *Tinotus morion*

Origin	Palaearctic, adventive in North America
Distribution	Canada: AB, BC, NB, NF, ON, QC, **SK**; USA: CT, NV
New records	New provincial record: **Saskatchewan:** Maple Creek, horse manure, 49.9037°, -109.5909°, 764 m, 2.IX.2012(BGC)1 male
References	Klimaszewski et al. 2002, 2005, 2011, Gouix and Klimaszewski 2007, Majka and Klimaszewski 2010

###### Natural history.

In Saskatchewan, one male was captured in horse manure. Elsewhere, adults were collected from decaying organic matter, fungi, animal droppings, human feces, and carrion (Klimaszewski et al. 2002). Larvae are parasitic on fly pupae (Klimaszewski et al. 2002). The adults were collected from June to September.

#### ATHETINI Casey

##### Acrotona recondita

Taxon classificationAnimaliaColeopteraStaphylinidae

(Erichson)

Figs 1–8

Acrotona recondita LECTOTYPE (male): *Homalota recondita* Erichson; USA: Pennsylv[ania], Zimm[erman] [on green rectangular card]; # 5472; Typus; *recondita* Er.; Lectotypus, male, *Homalota recondita* Erichson, V.I. Gusarov des. (not published); our lectotype designation label as *Homalota recondita*; *Acrotona recondita* (Er.) V.I. Gusarov 2002 (ZMB) studied. PARALECTOTYPES: labelled as the lectotype, our paralectotype designation label (ZMB) 1 male, 2 females, 1 sex undetermined, specimen partially damaged, studied.
Arisota apacheella Casey 1910: 135. Synonymized by Moore and Legner 1975: 371.
Arisota insueta Casey 1910: 134. Synonymized by Moore and Legner 1975: 371.
Arisota pomonensis Casey 1910: 135. Synonymized by Moore and Legner 1975: 371.
Arisota speculifer Casey 1910: 135. Synonymized by Moore and Legner 1975: 371.
Arisota tetricula Casey 1910: 134. Synonymized by Moore and Legner 1975: 371.
Arisota umbrina Casey 1910: 136. Synonymized by Moore and Legner 1975: 371.

###### Diagnosis.

**Figures 1–8.**
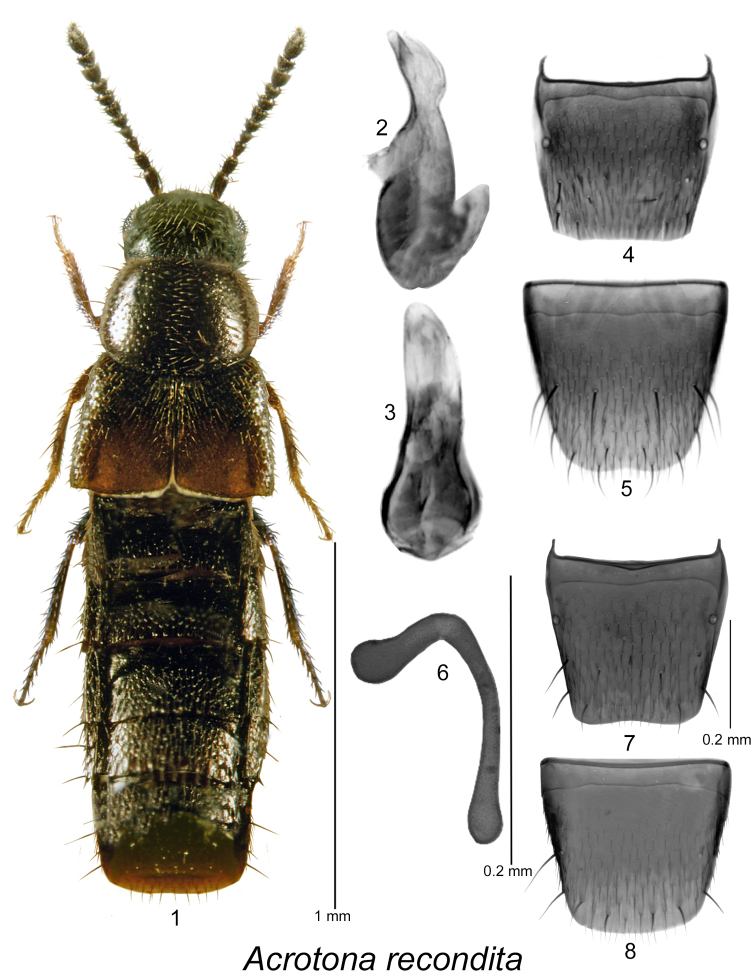
*Acrotona recondita* (Casey): **1** habitus in dorsal view **2** median lobe of aedeagus in lateral view, and **3** in dorsal view **4** male tergite VIII **5** male sternite VIII **6** spermatheca in lateral view **7** female tergite VIII **8** female sternite VIII; **1, 6–8** based on female from Saskatchewan **2–5** based on male from Pennsylvania.

Body narrowly subparallel (Fig. 1), length 1.7–1.8 mm, dark brown with two large reddish-brown spots on posterior sutural part of elytra and lighter colour tarsi (Fig. 1); head, pronotum and elytra coarsely and sparsely punctate, punctures large; pubescence sparse; integument strongly glossy; pronotum transverse, slightly narrower than elytra, pubescence directed laterad from median line; elytra at suture about as long as pronotum; abdomen subparallel. MALE. Median lobe of aedeagus with oval bulbus and narrowly elongate and rounded tubus in dorsal view (Fig. 3), in lateral view tubus slightly arcuate basally and straight apically (Fig. 2); internal sac structures not pronounced; tergite VIII truncate apically (Fig. 4); sternite VIII slightly emarginated at apex and with broad distance between base of disc and antecostal suture (Fig. 5). FEMALE. Tergite VIII truncate apically (Fig. 7); sternite VIII broadly arcuate apically (Fig. 8); spermatheca with narrowly elongate club-shaped capsule angularly connected to narrow and long stem, together forming L-shaped structure (Fig. 6).

###### Distribution.

**Table:** Distribution of *Acrotona recondita*

Origin	Nearctic
Distribution	Canada: first record for Canada and **SK**; USA: AR, CA, NH, NV, NY, PA
New records	New provincial record; **Saskatchewan:** Maple Creek, horse manure, 49.9037°, -109.5909°, 764 m, 2.IX.2012(BGC)1 female
References	Erichson 1839, Bland 1865, Casey 1910, Moore and Legner 1975

###### Natural history.

The single female in Saskatchewan was captured in horse manure.

###### Remarks.

This species was originally described by Erichson (1839) as *Homalota recondita*, from Pennsylvania. It clearly does not belong to *Homalota* and was subsequently listed by Moore and Legner (1975) as belonging to the subgenus *Dimetrota* of *Atheta*. Gusarov, V.I. identified types of *Homalota recondita* as *Acrotona*. The inclusion of this species in *Acrotona* needs confirmation because it has scarcly visible minute part of pronotal hypomeron visible in lateral view.

##### Aloconota sulcifrons

Taxon classificationAnimaliaColeopteraStaphylinidae

(Stephens)

Aloconota sulcifrons (for diagnosis and illustrations, see Klimaszewski et al. 2011)

###### Distribution.

**Table:** Distribution of *Aloconota sulcifrons*

Origin	Palaearctic, adventive in North America
Distribution	Canada: **MB**, NB, NF, ON, QC,; USA: AL, IL, IN, KY, MO, NH, NY, TN, VA, WV
New records	New provincial record: **Manitoba**, Winnipeg, Whittier Park, Red River bank dry litter, 49.8968, -97.1155, 226 m, 21.X.2009(BGC)1 female
References	Klimaszewski and Peck 1986 [under *Aloconota insecta*], Gusarov 2003a, Webster et al. 2009, Majka and Klimaszewski 2010, Bousquet et al. 2013

###### Natural history.

In Saskatchewan, one female was captured in dry litter on the banks of the Red River. In Newfoundland, adults were collected in mixedwood forest litter, in litter in riparian zones along forested streams, a sandy lakeshore and a marsh. Elsewhere, adults were recorded from organic debris, fungi and carrion, and often found in caves in the USA (Klimaszewski and Peck 1986, Klimaszewski et al. 2011). The adults were collected from June to October.

##### Atheta (Datomicra) celata

Taxon classificationAnimaliaColeopteraStaphylinidae

(Erichson)

Atheta (Datomicra) celata (for diagnosis and illustrations, see Klimaszewski et al. 2011)

###### Distribution.

**Table:** Distribution of Atheta (Datomicra) celata

Origin	Probably Holarctic
Distribution	Canada: BC, NB, NL, NS, QC, **SK**; USA: AK (as *Datomicra wrangleri* Casey)
New records	New provincial records: **Saskatchewan:** Prince Albert, sandy beach, 53.9804°, -106.28°, 532 m, 4.VI.2013 (BGC, LFC) 2 males, 1 female: Meadow Lake, wet spruce litter, 54.4144°, -108.8897°, 486 m, 7.VI.2013 (BGC, LFC) 1 male, 1 female
References	Casey 1910, Benick and Lohse 1974, Majka et al. 2006, Gouix and Klimaszewski 2007, Majka and Klimaszewski 2008, 2010, Klimaszewski et al. 2011, Bousquet et al. 2013

###### Natural history.

In Saskatchewan, adults were captured on a sandy beach and in wet spruce litter. In Newfoundland, one specimen was collected in a carrion-baited pitfall trap in a forest (Klimaszewski et al. 2011). In Nova Scotia, adults were collected in nests of boreal and saw-whet owls (Klimaszewski and Majka 2007). The adults were collected in July and August.

##### Atheta (Datomicra) dadopora

Taxon classificationAnimaliaColeopteraStaphylinidae

Thomson

Atheta (Datomicra) dadopora (for diagnosis and illustrations, see Klimaszewski et al. 2011; for synonyms, see Gusarov 2003a)

###### Distribution.

**Table:** Distribution of Atheta (Datomicra) dadopora

Origin	Probably Holarctic
Distribution	Canada: AB, **BC**, LB, NB, NF, NS, ON, PE, QC, **SK**, YT,; USA: AK, NY, PA, RI
New records	New provincial records: **Saskatchewan:** Meadow Lake, wet spruce litter, 54.4144°, -108.8897°, 486 m, 7.VI.2013 (BGC, LFC) 1 male, 1 female; Prince Albert, poplar/spruce litter, 53.9665°, -106.0652°, 538 m, 4.VI.2013(BGC)1 male; **British Columbia:** Liard River, bison scats, 59.4288°, -126.1157°, 468 m, 10.VI.2013 (BGC, LFC) 1 female, 1 male
References	Gusarov 2003a, Klimaszewski et al. 2005, 2011, Gouix and Klimaszewski 2007, Majka and Klimaszewski 2008, 2010, Bousquet et al. 2013

###### Natural history.

This species is strongly associated with forests. The habitats of adults include bison faeces in British Columbia and wet spruce litter and poplar-spruce litter in Saskatchewan. In Newfoundland, adults were collected using carrion traps and flight intercept traps in various mixedwood and coniferous forest types (Klimaszewski et al. 2011). Some specimens were found in rotting mushrooms in forests and under the bark of decaying spruce logs (Klimaszewski et al. 2011). Elsewhere in North America it was collected from fungi and in pitfall traps in forests. The adults were collected from June to August.

##### Atheta (Datomicra) nigra

Taxon classificationAnimaliaColeopteraStaphylinidae

(Kraatz)

Figs 9–15

###### Diagnosis.

**Figures 9–15.**
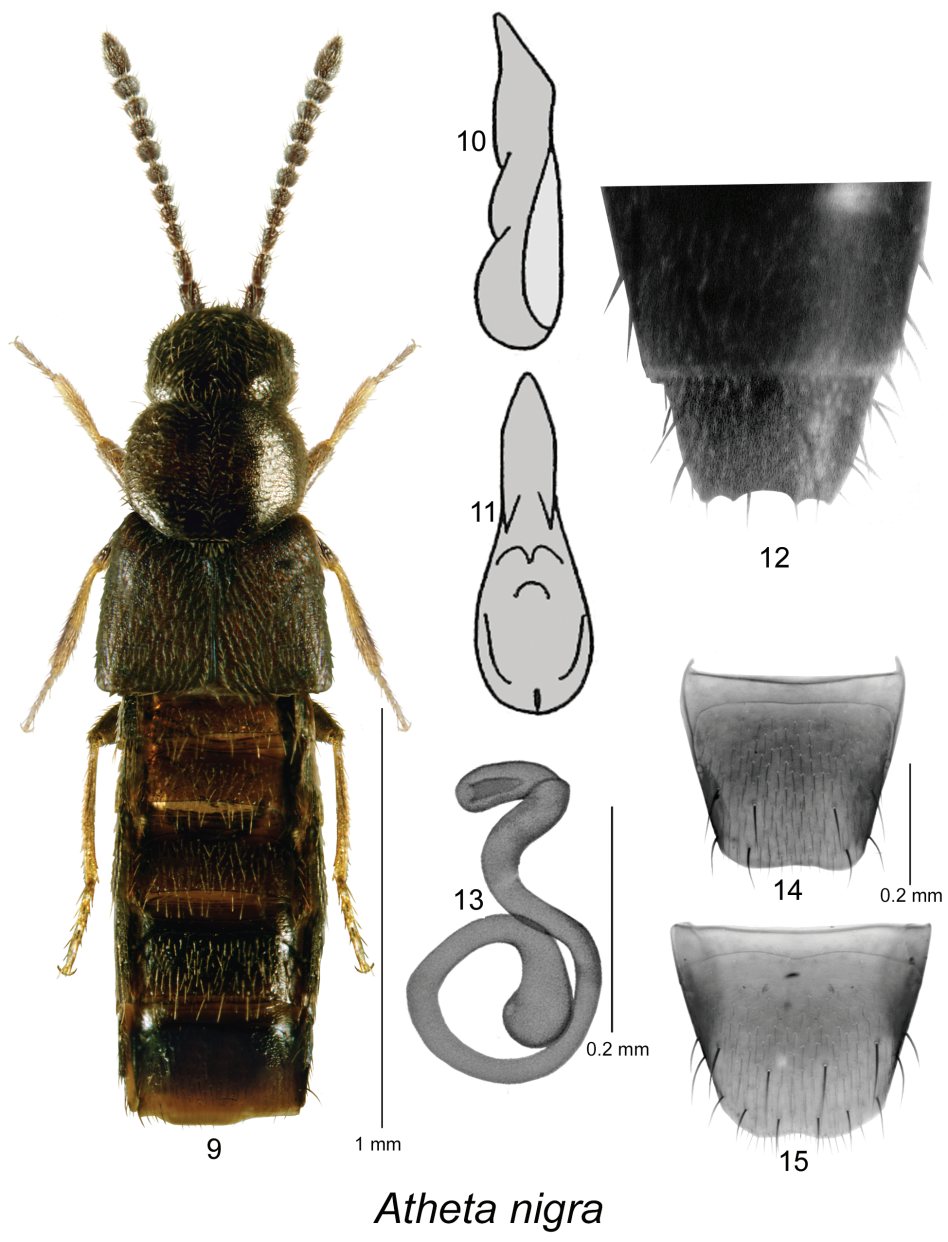
Atheta (Datomicra) nigra (Kraatz): **9** habitus in dorsal view **10** median lobe of aedeagus in lateral view, and **11** in ventral view **12** apical part of male abdomen with visible 4 dents on apical margin of male tergite VIII **13** spermatheca **14** female tergite VIII **15** female sternite VIII; **9, 13–15** based on a female from Saskatchewan **10, 11** after Benick and Lohse (1974) **12** based on a male from Germany.

Body narrowly elongate (Fig. 9), length 1.8–2.3 mm, dark brown to black, legs with at least tarsi reddish-brown; head, pronotum and elytra finely and densely punctate, punctures small; pubescence dense; integument strongly glossy; pronotum transverse, slightly narrower than elytra, with median line of disc well defined, pubescence directed laterad from median line; elytra at suture slightly longer than pronotum; abdomen subparallel. MALE. Median lobe of aedeagus with oval bulbus and narrowly elongate and pointed tubus in ventral view (Fig. 11), in lateral view tubus slightly sinuate and slightly pointed ventrally at apex (Fig. 10); internal sac structures not pronounced; tergite VIII with four small dents apically (Fig. 12); sternite VIII broadly rounded apically. FEMALE. Tergite VIII truncate and slightly concave apically (Fig. 14); sternite VIII broadly arcuate apically with shallow apical emargination (Fig. 15); spermatheca with narrowly elongate and angularly bent capsule bearing large and long apical invagination, stem narrow, and with a single posterior coil bearing swollen apical part (Fig. 13).

###### Distribution.

**Table:** Distribution of Atheta (Datomicra) nigra

Origin	Palaearctic, adventive in North America
Distribution	First record for **North America**, **Canada** and **SK**; USA unrecorded
New records	New country and provincial record: **Saskatchewan:** Maple Creek, horse manure, 49.9037°, -109.5909°, 764 m, 2.IX.2012(LFC)1 female
References	Kraatz 1856, Benick and Lohse 1974, Smetana 2004, Klimaszewski and Majka 2007

###### Natural history.

The single female in Saskatchewan was captured in horse manure in September.

###### Remarks.

This species is similar to our native Atheta (Datomicra) acadiensis Klimaszewski & Majka (2007) described from Nova Scotia but it is readily distinguishable by the morphology of genital structures. For illustrations of *Atheta acadiensis*, see Klimaszewski and Majka (2007).

##### Atheta (Bessobia) cryptica

Taxon classificationAnimaliaColeopteraStaphylinidae

(Lohse)

Atheta (Bessobia) cryptica (for diagnosis and illustrations, see Klimaszewski et al. 2011)

###### Distribution.

**Table:** Distribution of Atheta (Bessobia) cryptica

Origin	Nearctic
Distribution	Canada: **BC**, NF, QC, YT
New records	New provincial record: British Columbia, Summit Lake, willow/aspen litter, 58.6616°, -124.5215°, 1238 m, 10.VI.2013 (BGC, LFC) 2 males, 2 females
References	Lohse et al. 1990, Gouix and Klimaszewski 2007, Klimaszewski et al. 2008, 2011, Bousquet et al. 2013

###### Natural history.

In British Columbia, adults were captured in willow-aspen litter. In Newfoundland, adults were collected using pitfall traps in fir forests (Klimaszewski et al. 2011). In Yukon Territory, adults were collected from sifted willow litter (*Salix* sp.) (Klimaszewski et al. 2008). The adults were collected from May to July.

##### Atheta (Dimetrota) fanatica

Taxon classificationAnimaliaColeopteraStaphylinidae

Casey

Atheta (Dimetrota) fanatica (for diagnosis and illustrations, see Klimaszewski et al. 2011)

###### Distribution.

**Table:** Distribution of Atheta (Dimetrota) fanatica

Origin	Nearctic
Distribution	Canada: **AB**, BC [as *Atheta fanatica*], LB, NB, NS, QC [as *Atheta irrita*], **SK**; USA: AK, NV [as *Atheta irrita*]; likely transcontinental in Canada
New records	New provincial records: **Saskatchewan:** La Ronge, wet spruce litter, 55.118°, -105.2457°, 366 m, 6.VI.2013(BGC)1 female; **Alberta:** c. 100 km NW of Peace River, 56.68°, -118.63°, EMEND compartment 908, white spruce log in early decay stage, 12.VI.2012 (NoFC) 1 male
References	Casey 1910, 1911, Moore and Legner 1975, Campbell and Davies 1991, Majka et al. 2006, Gouix and Klimaszewski 2007, Webster et al. 2009, Majka and Klimaszewski 2010, Klimaszewski et al. 2011, Bousquet et al. 2013

###### Natural history.

In Saskatchewan, a female was captured in wet spruce litter, and one Newfoundland specimen was captured using a carrion-baited pitfall trap in a spruce/moss forest (Klimaszewski et al. 2011). In Alberta, one male was captured in an early decay stage of a white spruce log in spruce-aspen mixed forest. Elsewhere, adults were collected in the nests of several owl species, in maple forest, in oyster mushrooms (*Pleurotus* sp.), and in organic material on standing trees (Majka et al. 2006, Webster et al. 2009). The adults were collected from June to August.

##### Atheta (Atheta) graminicola

Taxon classificationAnimaliaColeopteraStaphylinidae

(Gravenhorst)

Atheta (Atheta) graminicola (for diagnosis and illustrations, see Klimaszewski et al. 2011)

###### Distribution.

**Table:** Distribution of Atheta (Atheta) graminicola

Origin	Holarctic
Distribution	Canada: AB, BC, LB, MB, NB, NF, NT, ON, QC, **SK**, YT; USA: AL, OR; Palaearctic: Europe, Asia
New records	New provincial record: **Saskatchewan:** Prince Albert, sandy beach, 53.9804°, -106.28°, 532 m, 4.VI.2013 (BGC, LFC) 1 male, 1 female
References	Lohse and Smetana 1985, Lohse et al. 1990 [as *Atheta granulata* Mannerheim], Gusarov 2003a, Gouix and Klimaszewski 2007, Webster et al. 2009, Majka and Klimaszewski 2010, Klimaszewski et al. 2011, Bousquet et al. 2013

###### Natural history.

In Saskatchewan, adults were captured on a sandy beach. In Newfoundland, some adults were collected using a flight intercept trap in a mixed forest (Klimaszewski et al. 2011). Elsewhere, adults occur in forest leaf litter, at edges of streams and pools, in moss and in drift material (Lohse et al. 1990, Webster et al. 2009). The adults were collected from April to June.

##### Atheta (Pseudota) klagesi

Taxon classificationAnimaliaColeopteraStaphylinidae

Bernhauer

Atheta (Pseudota) klagesi (for diagnosis and illustrations, see Klimaszewski et al. 2011)

###### Distribution.

**Table:** Distribution of Atheta (Pseudota) klagesi

Origin	Nearctic
Distribution	Canada: AB, BC, NB, NF, NS, PE, QC, ON, **SK**, YT; USA: IA, ME, MN, NJ, NY, PA
New records	New provincial record: **Saskatchewan:** Cypress Hills, near pond, riparian, 49.6704°, -109.5005°, 1189 m, 2.IX.2012(BGC)1 male
References	Gusarov 2003a, Klimaszewski et al. 2007b, 2011, Gouix and Klimaszewski 2007, Majka and Klimaszewski 2008, 2010, Webster et al. 2009, Bousquet et al. 2013

###### Natural history.

In Saskatchewan, one male was captured from the riparian zone of a pond. In Newfoundland, most adults were collected in forests of various types (deciduous, coniferous, mixedwood, riparian) using carrion-baited pitfall traps and flight intercept traps, as well as on coastal barrens using pitfall traps and on rotting mushrooms (Klimaszewski et al. 2011). Elsewhere, adults were collected on gilled, polypore and coral fungi, in compost and other organic debris, and in rotten logs, and the usual habitat is forest, e.g., hardwoods, eastern white cedar swamps, red spruce/yellow birch, hemlock, mixedwood (Klimaszewski and Peck 1986, Klimaszewski et al. 2005, 2007b, Majka and Klimaszewski 2008, Webster et al. 2009, Majka and Klimaszewski 2010). The adults were collected from April to August.

##### Atheta (Chaetida) longicornis

Taxon classificationAnimaliaColeopteraStaphylinidae

(Gravenhorst)

Atheta (Chaetida) longicornis (for diagnosis and illustrations, see Klimaszewski et al. 2011)

###### Distribution.

**Table:** Distribution of Atheta (Chaetida) longicornis

Origin	Palaearctic; adventive in North America
Distribution	Canada: NB, NF, NS, QC, **SK**; USA: CA, MN; Palaearctic: Europe, North Africa, Asia, and Oriental region
New records	New provincial record: **Saskatchewan:** Maple Creek, horse manure, 49.9037°, -109.5909°, 764 m, 2.IX.2012 (BGC, LFC) 2 males, 1 female
References	Klimaszewski et al. 2007a, 2011, Gouix and Klimaszewski 2007, Webster et al. 2009, Majka and Klimaszewski 2010, Michaud et al. 2010, Bousquet et al. 2013

###### Natural history.

In Saskatchewan, specimens were captured in horse manure. Elsewhere, adults are usually associated with cow dung, carrion, compost, rotting mushrooms, and other rotting organic substrates (Klimaszewski et al. 2007a, Webster et al. 2009, Michaud et al. 2010). The adults were collected from May to October.

##### Atheta (Microdota) platonoffi

Taxon classificationAnimaliaColeopteraStaphylinidae

Brundin

Atheta (Microdota) platonoffi (for diagnosis and illustrations, see Klimaszewski et al. 2011)

###### Distribution.

**Table:** Distribution of Atheta (Microdota) platonoffi

Origin	Holarctic
Distribution	Canada: AB, LB, NB, NF, NS, **SK**; USA: AK; Palaearctic: northern Europe.
New records	New provincial records: **Saskatchewan:** Prince Albert, ferns and scat, 53.987, -106.2802, 532 m (BGC) 1 female; Meadow Lake, wet spruce litter, 54.4144, -108.8897, 486 m, 7.VI.2013 (BGC, LFC) 2 males, 2 females
References	Klimaszewski et al. 2005, 2011, Gouix and Klimaszewski 2007, Majka and Klimaszewski 2008, 2010, Bousquet et al. 2013

###### Natural history.

In Saskatchewan, adults were found on ferns and scat, and in wet spruce litter. In Newfoundland, adults were collected using carrion-baited pitfall traps and flight intercept traps in various mixedwood and coniferous forest types (Klimaszewski et al. 2011). In New Brunswick, adults were captured from litter in a red spruce forest (Klimaszewski et al. 2005). The adults were collected from June to August.

##### Atheta (Dimetrota) prudhoensis

Taxon classificationAnimaliaColeopteraStaphylinidae

(Lohse)

Atheta (Dimetrota) prudhoensis (for diagnosis and illustrations, see Klimaszewski et al. 2011)

###### Distribution.

**Table:** Distribution of Atheta (Dimetrota) prudhoensis

Origin	Nearctic
Distribution	Canada: LB, NB, NF, NS, QC, ON, **SK**, YT; USA: AK, VT
New records	New provincial record: **Saskatchewan:** Maple Creek, horse manure, 49.9037°, -109.5909°, 764 m, 2.IX.2012(BGC)1 female
References	Lohse et al. 1990, Gusarov 2003a, Klimaszewski et al. 2007a, 2011, Gouix and Klimaszewski 2007, Majka and Klimaszewski 2008, 2010, Webster et al. 2009, Bousquet et al. 2013

###### Natural history.

In Saskatchewan, adults were found in horse manure. In Newfoundland, adults were collected using carrion-baited pitfall traps and flight intercept traps in conifer-dominated forests, including upland and riparian habitats (Klimaszewski et al. 2011). Elsewhere, adults were collected from gilled mushrooms, compost, and leaf litter in various forest types, e.g., birch, maple, oak, hemlock mixed forests and spruce forest (Lohse et al. 1990, Klimaszewski et al. 2007a, Webster et al. 2009). The adults were collected from June to September.

##### Atheta (Microdota) pseudosubtilis

Taxon classificationAnimaliaColeopteraStaphylinidae

Klimaszewski & Langor

Atheta (Microdota) pseudosubtilis (for diagnosis and illustrations, see Klimaszewski et al. 2011)

###### Distribution.

**Table:** Distribution of Atheta (Microdota) pseudosubtilis

Origin	Nearctic
Distribution	Canada: **AB**, LB, NB, NF, QC
New records	New provincial record: **Alberta:** c. 100 km NW of Peace River, Blk C31, 5.93 ha aggregated retention of white spruce, 56.68°, -118.64°, 21.VI.2011 (NoFC) 1 female
References	Klimaszewski et al. 2011, Bousquet et al. 2013

###### Natural history.

In Alberta, one female was found in a white spruce dominated aggregated retention patch (5.93 ha) surrounded by 10-year-old regenerating coniferous trees using a window trap attached to the trunk of white spruce snag. Elsewhere, adults were collected from unbaited and baited pitfall traps and flight intercept traps in various coniferous and mixedwood forest types (Klimaszewski et al. 2011). The adults were collected from June to August.

##### Atheta (Alaobia) ventricosa

Taxon classificationAnimaliaColeopteraStaphylinidae

Bernhauer

Atheta (Alaobia) ventricosa (for diagnosis and illustrations, see Klimaszewski et al. 2011)

###### Distribution.

**Table:** Distribution of Atheta (Alaobia) ventricosa

Origin	Nearctic
Distribution	Canada: AB, BC, LB, NB, NF, NS, ON, **SK**, YT; USA: AK, DC, NC, NJ, NY, PA, VT
New records	New provincial record: **Saskatchewan:** Cypress Hills, near pond, riparian, 49.6704°, -109.5005°, 1189 m, 2.IX.2012(BGC)1 male
References	Gusarov 2003a, Klimaszewski et al. 2005, 2008, 2011, Gouix and Klimaszewski 2007, Majka and Klimaszewski 2008, 2010, Bousquet et al. 2013

###### Natural history.

In Saskatchewan, one male was captured in the riparian zone of a pond. In Newfoundland, adults were collected in unbaited and carrion-baited pitfall traps and flight intercept traps in various coniferous and mixedwood forest types (Klimaszewski et al. 2011). Elsewhere, adults were found in coniferous forests and in organic litter in mixed forests (Klimaszewski et al. 2005, Majka and Klimaszewski 2008).The adults were collected from May to October.

##### Boreophilia davidgei

Taxon classificationAnimaliaColeopteraStaphylinidae

Klimaszewski & Godin

Boreophilia davidgei (for diagnosis and illustrations, see Klimaszewski et al. 2012)

###### Distribution.

**Table:** Distribution of *Boreophilia davidgei*

Origin	Nearctic
Distribution	Canada: **AB**, YT
New records	New provincial records: **Alberta:** c. 100 km NW of Peace River, Blk C14, 2.93 ha aggregated retention, white spruce girdled in 2010, 56.712°, -118.779°, 13.IX.2011 (NoFC) 1 female; Block C14, 1.43 ha aggregated retention, white spruce snag, 56.7103°, -118.7786°, 21.VI.2010 (NoFC) 1 female; Block C31, 1.71 ha aggregated retention, white spruce snag, 56.688°, -118.645°, 20.VI.2010 (NoFC) 1 female; Block C31, white spruce intact forest, white spruce snag, 56.682°, -118.636°, 15.VIII.2010 (NoFC) 1 male; EMEND compartment 896, white spruce snag, 56.7571°, -118.3981°, 810.9 m, 10.VII.2010 (NoFC) 1 female; EMEND compartment 919, regenerating aspen (*Populus tremuloides* Michaux), 56.7964°, -118.3607°, 715 m, 18.VI.2010 (NoFC) 1 female
References	Klimaszewski et al. 2012, Bousquet et al. 2013

###### Natural history.

In Alberta, adults were collected using window traps installed on the trunk of a recently girdled white spruce tree and on snags. The adults were found in white spruce dominated aggregated retention patches (> 1.43 ha) surrounded by 10-year-old coniferous regenerating matrix, small aggregated retention patch (0.20 ha) surrounded by 20% dispersed retention, 10-year-old regenerating aspen stand, and intact white spuce forest. In Yukon Territory, adults were collected using pitfall traps and sifting organic litters in various coniferous and mixedwood forest types (Klimaszewski et al. 2012). The adults were collected from May to September.

##### Boreophilia islandica

Taxon classificationAnimaliaColeopteraStaphylinidae

(Kraatz)

Boreophilia islandica (for diagnosis and illustrations, see Klimaszewski et al. 2011)

###### Distribution.

**Table:** Distribution of *Boreophilia islandica*

Origin	Holarctic
Distribution	Canada: **AB**, NF, NT, NU, YT; USA: AK; Palaearctic: Europe, Asia
New records	New provincial records: **Alberta:** c. 90 km NW of Peace River, EMEND compartment 919, white spruce log in intermediate decay stage, 56.7968°, -118.3603°, 715 m, 18.VI.2010 (NoFC) 1 female; EMEND compartment 892, regenerating aspen (*Populus tremuloides* Michaux), 56.7506°, -118.3994°, 781.1 m, 18.VI.2010 (NoFC) 1 female
References	Lohse et al. 1990, Gouix and Klimaszewski 2007, Klimaszewski et al. 2011, Bousquet et al. 2013

###### Natural history.

In Alberta, one female was collected in June in an aggregated retention patch (0.46 ha) surrounded by 20% dispersed retention of a white spruce dominated stand, using an emergence trap attached to the trunk of intermediate decay stage of white spruce log. The other female was captured in 10-year-old regenerating trembling aspen, using a window trap. This is the first habitat record of the species.

##### Boreostiba parvipennis

Taxon classificationAnimaliaColeopteraStaphylinidae

(Bernhauer)

Boreostiba parvipennis (for diagnosis and illustrations, see Klimaszewski et al. 2011)

###### Distribution.

**Table:** Distribution of *Boreostiba parvipennis*

Origin	Nearctic
Distribution	Canada: AB, LB, NF, **NT**, QC, YT; USA: AK, NH
New records	New provincial records: **Northwest Territories:** 27 km west of Yellowknife, aspen, 62.522°, -114.8859°, 171 m, 1.V.2009 (BGC, LFC) 1 male 1 female; 32 km west of Yellowknife, birch/spruce, 62.531°, -114.9581°, 168 m, 3.VI.2009(LFC)1 female
References	Lohse et al. 1990 [as *Boreostiba hudsonica*], Gusarov 2003a, Gouix and Klimaszewski 2007, Klimaszewski et al. 2008, 2011, Bousquet et al. 2013

###### Natural history.

In the Northwest Territories, adults were found in aspen and birch-spruce litter. In Newfoundland, adults were collected mostly using pitfall traps in fir and spruce forests (Klimaszewski et al. 2011). One specimen was found in a rotting mushroom and another under detritus on a sandy beach (Klimaszewski et al. 2011). The adults were collected from May to August.

##### Dinaraea pacei

Taxon classificationAnimaliaColeopteraStaphylinidae

Klimaszewski & Langor

Dinaraea pacei (for diagnosis and illustrations, see Klimaszewski et al. 2011)

###### Distribution.

**Table:** Distribution of *Dinaraea pacei*

Origin	Nearctic
Distribution	Canada: AB, BC, LB, NB, NF, QC, **YT**; USA: AK
New records	New provincial record: **Yukon Territory**, EMAN, Fireweed Dr., Lindgren 2 weeks; white spruce, 60.6014°, -134.9387°, 772 m, 12.VI.2013(BGC)1 female
References	Klimaszewski et al. 2011, 2013, Bousquet et al. 2013

###### Natural history.

One female was captured in the Yukon using a Lindgren funnel trap in a white spruce stand. Adults in Newfoundland and Labrador were collected using pitfall traps and flight intercept traps in various coniferous forest types, and one specimen was collected under the bark of a dead red pine (Klimaszewski et al. 2011). In British Columbia, adults were caught in emergence traps attached to the trunks of lodgepole pine (*Pinus contorta* Dougl. ex Loud. *latifolia* Engelm.) infested by mountain pine beetle (*Dendroctonus ponderosae* Hopkins) (Langor, unpublished). In New Brunswick, adults were found: under the bark of large fallen spruce in an old-growth eastern white cedar swamp; under tight bark of American elm; in a silver maple forest; in fleshy polypore fungi at the base of a dead standing *Populus* sp. in a wet alder swamp; in a group of *Pholiota* sp. at the base of a dead *Populus* sp. in a mixed forest. In Quebec, adults were found in dead black spruce in a black spruce forest. Adults were also captured in Lindgren funnel traps deployed in an old-growth white spruce (*Picea glauca* (Moench) Voss) and balsam fir forest, an old mixed forest with red and white spruce, red and white pine (*Pinus strobus* L.), and a rich Appalachian hardwood forest with some conifers (Klimaszewski et al. 2013). The adults were collected from March to September.

##### Dinaraea worki

Taxon classificationAnimaliaColeopteraStaphylinidae

Klimaszewski & Jacobs

Dinaraea worki (for diagnosis and illustrations, see Klimaszewski et al. 2013)

###### Distribution.

**Table:** Distribution of *Dinaraea worki*

Origin	Holarctic
Distribution	Canada: **AB**, QC
New records	New provincial records: **Alberta:** c. 90 km NW of Peace River, EMEND compartment 922, white spruce girdled in 2009, 56.7971°, -118.3750°, 17.IX.2009 (NoFC) female; EMEND compartment 918, white spruce log in intermediate decay stage, 56.792°, -118.364°, 757.8 m, 18.VI.2010 (NoFC) 1 female; EMEND compartment 932, white spruce snag, 56.8068°, -118.3290°, 17.VII.2009 (NoFC) 1 female; EMEND compartment 911, white spruce log in early decay stage, 4.VIII.2011 (NoFC) 1 female
Reference	Klimaszewski et al. 2013

###### Natural history.

In Alberta, adults were collected using window traps attached to a girdled white spruce tree and a snag, and were reared from white spruce logs in early and intermediate decay stages. In Quebec, adults were found in dead and dying black spruce (*Picea mariana* Mill. (BSP)) in black spruce dominated stands. The adults were collected from June to September.

##### Liogluta aloconotoides

Taxon classificationAnimaliaColeopteraStaphylinidae

Lohse

Liogluta aloconotoides (for diagnosis and illustrations, see Klimaszewski et al. 2011)

###### Distribution.

**Table:** Distribution of *Liogluta aloconotoides*

Origin	Nearctic
Distribution	Canada: **AB**, LB, NB, NF, NS, YT
New records	New provincial records: **Alberta:** c. 90 km NW of Peace River, EMEND compartment 918, white spruce log in advanced decay stage, 56.792°, -118.364°, 757.8 m, 27.VIII.2009 and 9.VII.2010 (NoFC) 2 males
References	Klimaszewski et al. 2011, Webster et al. 2012, Bousquet et al. 2013

###### Natural history.

In Alberta, adults were reared from well-decayed white spruce logs. Elsewhere, adults were captured in various forest types including a recently burned forest. The adults were collected from July to October.

##### Lypoglossa franclemonti

Taxon classificationAnimaliaColeopteraStaphylinidae

Hoebeke

Lypoglossa franclemonti (for diagnosis and illustrations, see Klimaszewski et al. 2011)

###### Distribution.

**Table:** Distribution of *Lypoglossa franclemonti*

Origin	Nearctic
Distribution	Canada: AB, MB, NB, NF, NS, NT, QC, ON, **SK**, YT; USA: NY, VT
New records	New provincial record: **Saskatchewan:** Prince Albert, sandy beach, 53.9804°, -106.28°, 532 m, 2013.VI.3(BGC)1 male
References	Hoebeke 1992, Gusarov 2004, Gouix and Klimaszewski 2007, Webster et al. 2009, Majka and Klimaszewski 2010, Klimaszewski et al. 2011, Bousquet et al. 2013

###### Natural history.

In Saskatchewan, one male was captured on a sandy beach. In Newfoundland, a single specimen was collected in a pitfall trap in a balsam fir stand (Klimaszewski et al. 2011). Elsewhere, adults were captured in litter in spruce bogs, birch bogs, in moss and lichens, and in coniferous and deciduous forests (Gusarov 2004). The adults were collected from May to September.

##### Philhygra botanicarum

Taxon classificationAnimaliaColeopteraStaphylinidae

(Muona)

Philhygra botanicarum (for diagnosis and illustrations, see Klimaszewski et al. 2011)

###### Distribution.

**Table:** Distribution of *Philhygra botanicarum*

Origin	Holarctic
Distribution	Canada: BC, LB, NB, NF, NS, ON, **SK**, YT; Palaearctic: northern Europe
New records	New provincial record: **Saskatchewan:** Cypress Hills, wet willow stand, 49.5978°, -109.9231°, 1134 m, 2.IX.2012(BGC)1 male
References	Muona 1983, 1984, Gouix and Klimaszewski 2007, Klimaszewski et al. 2008, 2011, Webster et al. 2009, Majka and Klimaszewski 2010, Bousquet et al. 2013

###### Natural history.

In Saskatchewan, one male was captured in wet willow stand. In Newfoundland, adults were collected using flight intercept traps in mixedwood and coniferous forests, and along the margins of streams (Klimaszewski et al. 2011). In New Brunswick, adults were found on muddy soil, near margins of water in alder swamps, in mixed forests, in drift material on a lakeshore, and in moist leaves under a sap flow from a yellow birch (Webster et al. 2009). The adults were collected from May to August.

##### Philhygra clemens

Taxon classificationAnimaliaColeopteraStaphylinidae

(Casey)

Philhygra clemens (for details and body image, see Klimaszewski et al. 2005, 2007b, Majka and Klimaszewski 2008)

###### Distribution.

**Table:** Distribution of *Philhygra clemens*

Origin	Nearctic
Distribution	Canada: **MB**, NB, NS, QC, ON, YT; USA: WI
New records	New provincial record: **Manitoba:** Winnipeg, Whittier Park, Red River bank litter, 49.8996, -97.1250, 228 m, 18.X.2009(BGC)1 male
References	Casey 1910, Moore and Legner 1975, Klimaszewski et al. 2005, 2007b, Majka and Klimaszewski 2008, Bousquet et al. 2013

###### Natural history.

The Manitoba male was captured in litter on the bank of the Red River. In New Brunswick, it was found in red spruce (*Philhygra rubens*) forests (Klimaszewski et al. 2005), whereas in Quebec it was found in yellow birch (*Boreostiba alleghaniensis*) forests (Klimaszewski et al. 2007b). The adults were collected from June to October.

##### Philhygra jarmilae

Taxon classificationAnimaliaColeopteraStaphylinidae

Klimaszewski & Langor

Philhygra jarmilae (for diagnosis and illustrations, see Klimaszewski et al. 2011)

###### Distribution.

**Table:** Distribution of *Philhygra jarmilae*

Origin	Nearctic
Distribution	Canada: NB, NF, ON, **SK**, YT
New records	New provincial records: **Saskatchewan:** Meadow Lake, birch/alder litter, 54.4188°, -108.944°, 482 m, 7.VI.2013, 1 male (BGC) 1 female; Prince Albert, sandy beach, 53.9804°, -106.28°, 532 m, 4.VI.2013 (BGC, LFC) 1 male, 3 females; Cypress Hills, near pond, riparian, 49.5978°, -109.9231°, 1189 m, 2.IX.2012(LFC)1 male
References	Klimaszewski et al. 2011, Bousquet et al. 2013

###### Natural history.

The holotype was captured in a flight intercept trap in a mixedwood forest in Newfoundland (Klimaszewski et al. 2011). In Saskatchewan, adults were found in birch-alder litter, on a sandy beach, and in the riparian zone of a pond. The adults were collected from June to September.

##### Philhygra ripicoloides

Taxon classificationAnimaliaColeopteraStaphylinidae

Lohse

Philhygra ripicoloides (for diagnosis and illustrations, see Klimaszewski et al. 2011)

###### Distribution.

**Table:** Distribution of *Philhygra ripicoloides*

Origin	Nearctic
Distribution	Canada: NF, NT, **SK**, YT
New records	New provincial record: **Saskatchewan:** Prince Albert, sandy beach, 53.9804°, -106.28°, 532 m, 4.VI.2013(BGC)1 male
References	Lohse et al. 1990, Gouix and Klimaszewski 2007, Klimaszewski et al. 2011, Bousquet et al. 2013

###### Natural history.

In Saskatchewan, one male was captured on a sandy beach. Adults were collected from May to August (Klimaszewski et al 2011).

##### Philhygra rostrifera

Taxon classificationAnimaliaColeopteraStaphylinidae

Lohse

Philhygra rostrifera (for diagnosis and illustrations, see Klimaszewski et al. 2011)

###### Distribution.

**Table:** Distribution of *Philhygra rostrifera*

Origin	Nearctic
Distribution	Canada: NF, **NT**, **SK**, YT
New records	New provincial records: **Northwest Territories:** 32 km west of Yellowknife, birch/spruce, 62.531°, -114.9581°, 168 m, 3.VI.2009(BGC)1 male; **Saskatchewan:** Cypress Hills, wet willow stand, 49.5978°, -109.923°1, 1134 m, 2.IX.2012(LFC)1 male; Lug Creek, spruce/alder litter, 55.1776°, -106.6885°, 406 m, 6.VI.2013(BGC)1 female; Prince Albert, ferns and scat, 53.9804°, -106.28°, 532 m, 3.VI.2013(BGC)1 female; La Ronge, ditch litter in deciduous forest, 55.118°, -105.2457°, 366 m, 6.VI.2013(LFC)1 female
References	Lohse et al. 1990, Gouix and Klimaszewski 2007, Klimaszewski et al. 2011, Bousquet et al. 2013

###### Natural history.

In Northwest Territories, one male was captured in birch/spruce forest. In Saskatchewan, adults were found in wet willow thicket, spruce-alder litter, ditch litter in a deciduous forest, and on ferns and in scat. In Newfoundland, a single adult was collected from treading vegetation and sphagnum moss in a boggy area (Klimaszewski et al. 2011). In Yukon Territory, the species was found in moss in a meadow (Klimaszewski et al. 2012). The adults were collected from June to September.

##### Philhygra sinuipennis

Taxon classificationAnimaliaColeopteraStaphylinidae

Klimaszewski & Langor

Philhygra sinuipennis (for diagnosis and illustrations, see Klimaszewski et al. 2011)

###### Distribution.

**Table:** Distribution of *Philhygra sinuipennis*

Origin	Nearctic
Distribution	Canada: LB, NB, NF, **SK**, YT
New records	New provincial record: **Saskatchewan:** Prince Albert, sandy beach, 53.9804°, -106.28°, 532 m, 4.VI.2013(LFC)1 male
References	Klimaszewski et al. 2011, 2012, Bousquet et al. 2013

###### Natural history.

In Saskatchewan, one male was captured on a sandy beach. In Newfoundland, one specimen was collected amongst litter and stones on the sandy shore of a lake (Klimaszewski et al. 2011). The adults were collected in June and July.

##### Philhygra terrestris

Taxon classificationAnimaliaColeopteraStaphylinidae

Klimaszewski & Godin

Philhygra terrestris (for diagnosis and illustrations, see Klimaszewski et al. 2012)

###### Distribution.

**Table:** Distribution of *Philhygra terrestris*

Origin	Nearctic
Distribution	Canada: **SK**, YT
New records	New provincial records: **Saskatchewan:** Cypress Hills, wet willow stand, 49.5978°, -109.9231°, 1134 m, 2.IX.2012 (BGC, LFC) 2 males; Meadow Lake, birch/alder litter, 54.4188°, -108.944°, 482 m, 7-VI-2013, 2 females; La Ronge, alder/spruce litter, 55.118°, -105.2457°, 366 m, 6.VI.2013(BGC)1 female
References	Klimaszewski et al. 2012, Bousquet et al. 2013

###### Natural history.

In Saskatchewan, adults were captured in a wet willow stand and in birch-alder and birch-spruce litter. The Yukon specimen was collected from ground litter (Klimaszewski et al. 2012). The adults were collected from June to September.

##### Schistoglossa campbelli

Taxon classificationAnimaliaColeopteraStaphylinidae

Klimaszewski

Schistoglossa campbelli (for diagnosis and illustrations, see Klimaszewski et al. 2009a)

###### Distribution.

**Table:** Distribution of *Schistoglossa campbelli*

Origin	Nearctic
Distribution	Canada: **AB**, BC
New records	New provincial record: **Alberta:** c. 90 km NW of Peace River, EMEND compartment 932, white spruce snag, 56.8071°, -118.3276°, 6.VIII.2009 (NoFC) 1 female; same except, EMEND compartment 933, white spruce girdled in 2009, 56.8056°, -118.3328°, 19.VI.2010 (NoFC) 1 female
References	Klimaszewski et al. 2009a, Bousquet et al. 2013

###### Natural history.

In Alberta, adults were captured in window traps attached to a recent white spruce snag. In British Columbia, adults were captured by treading *Sphagnum* and *Carex* at the edge of a marsh (Klimaszewski et al. 2009a). The adults were collected in July and August.

##### Schistoglossa carexiana

Taxon classificationAnimaliaColeopteraStaphylinidae

Klimaszewski

Schistoglossa carexiana (for diagnosis and illustrations, see Klimaszewski et al. 2009a)

###### Distribution.

**Table:** Distribution of *Schistoglossa carexiana*

Origin	Nearctic
Distribution	Canada: BC, **SK**
New records	New provincial record: **Saskatchewan:** Prince Albert, sandy beach, 53.9804°, -106.28°, 532 m, 4.VI.2013(LFC)1 female
References	Klimaszewski et al. 2009a, Bousquet et al. 2013

###### Natural history.

In Saskatchewan, one female was captured on a sandy beach. In British Columbia, adults were captured by treading *Sphagnum* and *Carex* at the edge of a marsh (Klimaszewski et al. 2009a). The adults were collected from June to August.

##### Schistoglossa hampshirensis

Taxon classificationAnimaliaColeopteraStaphylinidae

Klimaszewski

Schistoglossa hampshirensis (for diagnosis and illustrations, see Klimaszewski et al. 2009a)

###### Distribution.

**Table:** Distribution of *Schistoglossa hampshirensis*

Origin	Nearctic
Distribution	Canada: **AB**, NB, QC; USA: NH
New records	New provincial record: **Alberta:** c. 100 km NW of Peace River, Block C14, 1.43 ha aggregated retention, white spruce girdled in 2010, 56.7103°, -118.7786°, 22.VI.2011 (NoFC) 1 female
References	Klimaszewski et al. 2009a, Bousquet et al. 2013

###### Natural history.

In Alberta, one female was captured in a window trap attached to a recently girdled white spruce tree. Elsewhere, adults were captured in *Salix*, *Vaccinium* and *Chamaedaphne* leaf litter (Klimaszewski et al. 2009a). The adults were collected from June to September.

##### Seeversiella globicollis

Taxon classificationAnimaliaColeopteraStaphylinidae

(Bernhauer)

Seeversiella globicollis (for diagnosis and illustrations, see Klimaszewski et al. 2011)

###### Distribution.

**Table:** Distribution of *Seeversiella globicollis*

Origin	Nearctic
Distribution	Canada: AB, BC, NF, NS, ON, QC, **SK**; USA: AZ, CO, ID, MN, MT, NH, SD, WI; Mexico; Guatemala
New records	New provincial record: **Saskatchewan:** Prince Albert, aspen stand, 54.7217°, -105.689°6, 484 m, 5.VI.2013(BGC)1 male
References	Ashe 1986, Gusarov 2003a,b, Gouix and Klimaszewski 2007, Majka and Klimaszewski 2008, 2010, Klimaszewski et al. 2011, Bousquet et al. 2013

###### Natural history.

In Saskatchewan, one male was captured in an aspen stand. In Newfoundland, adults were collected using pitfall traps in fir and riparian forests (Klimaszewski et al. 2011). Elsewhere, adults were found in leaf litter near a body of water, in litter near the sea and in mountain forests (Klimaszewski et al. 2011). The adults were collected from March to September.

##### Trichiusa pilosa

Taxon classificationAnimaliaColeopteraStaphylinidae

Casey

Figs 16–23

Trichiusa pilosa Casey 1894: 341, 343; Moore and Legner 1975: 504. LECTOTYPE (female): USA: RI [Rhode Island; in orig. descrip. Boston Neck]; Casey determ. *pilosa*-3; Casey bequest 1925; *Trichiusa pilosa* Casey, Gusarov V.I. det. 2010; our lectotype designation label, present designation (USNM). PARALECTOTYPES: labelled as the lectotype except: Casey determ. *pilosa*-4(USNM)1 male; Casey determ. *pilosa*-5(USNM)1 female; Type USNM 39424(USNM)1 male.
Trichiusa atra Casey 1906: 330; Moore and Legner 1975: 504. **New Synonymy.** LECTOTYPE (male): USA: McPherson, W. Kansas; *atra* Casey; Type USNM 39426; Casey bequest 1925; Lectotypus *Trichiusa atra* Casey, Gusarov V.I. det. 2011 [unpublished designation]; our lectotype designation label, present designation (USNM).
Trichiusa monticola Casey 1906: 328; Moore and Legner 1975: 504. **New Synonymy.** LECTOTYPE (male): USA: Coeur d’Alene, Idaho [in orig. descrip. H.F. Wickham]; *monticola* Casey; Type USNM 39421; Lectotypus *Trichiusa monticola* Casey, Gusarov, V.I. des. 2011[unpublished designation]; our lectotype designation label, present designation (USNM).
Trichiusa parviceps Casey 1906: 328; Moore and Legner 1975: 504. **New Synonymy.** LECTOTYPE (female): USA: Cin. [in orig. descript.: Ohio, Cincinnati, Chas. Dury]; *parviceps* Casey; Casey bequest 1925; Lectotypus *Trichiusa parviceps*, Gusarov V.I. des. 2011 [unpublished designation]; our lectotype designation label, present designation (USNM).
Trichiusa postica Casey 1906: 330; Moore and Legner 1975: 504. **New synonymy.** LECTOTYPE (male): W.H.H. [W.H. Harrington], Ottawa, Canada; Type USNM 39427; Casey bequest 1925; *Trichiusa pilosa* Casey, Gusarov, V.I. det. 2010; our lectotype designation label, present designation (USNM).

###### Diagnosis.

**Figures 16–23.**
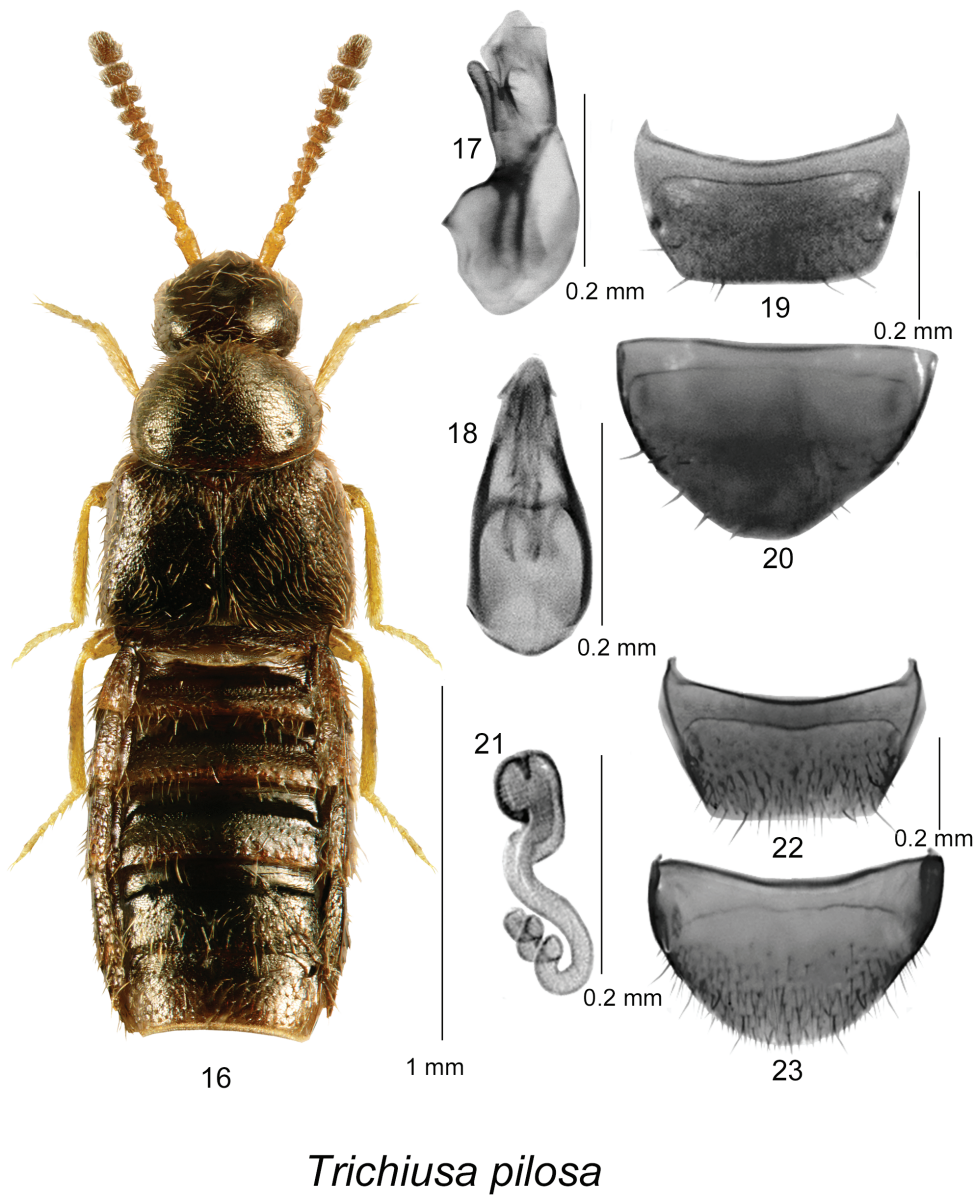
*Trichiusa pilosa* Casey: **16** habitus in dorsal view **17** median lobe of aedeagus in lateral view, and **18** in dorsal view **19** male tergite VIII **20** male sternite VIII **21** spermatheca in lateral view **22** female tergite VIII **23** female sternite VIII **17–23** based on type material from Rhode Island, USA.

Body broadly oval (Fig. 16), length 1.5–1.8 mm, dark brown with reddish tinge and slightly paler base of abdomen or uniformly black, appendages usually lighter than rest of body (Fig. 16); sparsely punctate and pubescent; setae straight and erect, particularly on pronotum; integument strongly glossy; head slightly narrower than pronotum; pronotum moderately transverse, rounded laterally and basally, distinctly narrower than elytra; elytra broad at suture about as long as pronotum; abdomen arcuate laterally and broadest at middle of its length. MALE. Median lobe of aedeagus with oval bulbus and triangularly shaped tubus in dorsal view (Fig. 18), in lateral view tubus slightly sinuate and narrow apically with apex narrowly rounded (Fig. 17); internal sac structures not pronounced; tergite VIII short and truncate apically (Fig. 19); sternite VIII slightly produced apically and rounded at apex and with narrow distance between base of disc and antecostal suture (Fig. 20). FEMALE. Tergite VIII short and truncate apically (Fig. 22); sternite VIII broadly arcuate apically (Fig. 23); spermatheca with broad and sac-shaped capsule with minute apical invagination, stem narrow, sinuate and narrowly twisted apically (Fig. 21).

###### Distribution.

**Table:** Distribution of *Trichiusa pilosa*

Origin	Nearctic
Distribution	Canada: **AB**, **BC**, NS, ON (as *Trichiusa postica*); USA: ID, IN, KS, OH, RI
New records	New provincial records: **Alberta:** c. 100 km NW of Peace River, Block C14, white spruce regenerating stand, 56.7079°, -118.7775°, 14.IX.2011 (NoFC) 1 female; **British Columbia:** Liard River, bison scats, 59.4288°, -126.1157°, 468 m, 10.VI.2013 (BGC, LFC) 1 male, 1 female
References	Casey 1894, 1906, Moore and Legner 1975, Majka and Klimaszewski 2010 (as *Trichiusa postica*), Brunke et al. 2012, Bousquet et al. 2013 (as *Trichiusa postica*)

###### Natural history.

In Alberta, one female was collected using a window trap deployed in a 10-year-old regenerating white spruce stand. The British Columbia specimens were found on bison scat. The adults were collected from June to September.

###### Comments.

The two specimens from British Columbia agree with the type series in external morphology and shape of genitalia except for the entirely black body. It is well known that many aleocharine species exhibit darker colour at higher latitudes compared with more southern populations.

#### GYMNUSINI Heer

##### Gymnusa campbelli

Taxon classificationAnimaliaColeopteraStaphylinidae

Klimaszewski

Gymnusa campbelli (for diagnosis and illustrations, see Klimaszewski 1979, Klimaszewski et al. 2011)

###### Distribution.

**Table:** Distribution of *Gymnusa campbelli*

Origin	Nearctic
Distribution	Canada: NB, NF, NT, ON, QC, **SK**, YT; USA: AK
New records	New provincial record: **Saskatchewan:** Prince Albert, sandy beach, 53.9804°, -106.28°, 532 m, 4.VI.2013(BGC)1 female
References	Klimaszewski 1979, Gouix and Klimaszewski 2007, Majka and Klimaszewski 2010, Klimaszewski et al. 2011, Bousquet et al. 2013

###### Natural history.

In Saskatchewan, one female was captured on a sandy beach. In Newfoundland, adults were collected in riparian areas (Klimaszewski et al. 2011). Elsewhere, adults were collected by treading wet moss on muddy shores of shallow lakes (Klimaszewski et al. 2011). The adults were collected from June to August.

#### HOMALOTINI Heer

##### Gyrophaena criddlei

Taxon classificationAnimaliaColeopteraStaphylinidae

Casey

Gyrophaena criddlei (for diagnosis and illustrations, see Klimaszewski et al. 2011)

###### Distribution.

**Table:** Distribution of *Gyrophaena criddlei*

Origin	Nearctic
Distribution	Canada: LB, MB, NB, ON, **SK**, YT
New records	New provincial record: **Saskatchewan:** Cypress Hills, mushroom, pine forest, 49.669°, -109.4998°, 1196 m, 2.IX.2012 (BGC, LFC) 2 males
References	Seevers 1951, Klimaszewski et al. 2009b, 2011, 2012, Majka and Klimaszewski 2010, Brunke et al. 2012, Bousquet et al. 2013

###### Natural history.

In Saskatchewan, adults were captured on a mushroom in pine forest. In Newfoundland, adults were collected using flight intercept traps in spruce-poplar forest (Klimaszewski et al. 2011). Elsewhere, adults were collected from gilled mushrooms in pine, hardwood and mixedwood forests (Seevers 1951, Klimaszewski et al. 2009b). The adults were collected in August and September.

##### Gyrophaena insolens

Taxon classificationAnimaliaColeopteraStaphylinidae

Casey

Gyrophaena insolens (for diagnosis and illustrations, see Seevers 1951, Klimaszewski et al. 2011)

###### Distribution.

**Table:** Distribution of *Gyrophaena insolens*

Origin	Nearctic
Distribution	Canada: BC, LB, MB, NB, NF, ON, **SK**; USA: MI
New records	New provincial record: **Saskatchewan:** Cypress Hills, mushroom, pine forest, 49.669°, -109.4998°, 1196 m, 2.IX.2012 (BGC, LFC) 2 males, 2 females
References	Seevers 1951, Klimaszewski et al. 2009b, 2011, Majka and Klimaszewski 2010, Brunke et al. 2012, Bousquet et al. 2013

###### Natural history.

In Saskatchewan, adults were collected from mushrooms in a pine forest. In Labrador, adults were collected using flight intercept traps in spruce-birch and spruce-poplar forests (Klimaszewski et al. 2011). Elsewhere, adults were collected from gilled mushrooms (*Russula* sp.) in mixed forests, white and red spruce forests, white cedar swamps, yellow birch and spruce forests, and oak and maple forests (Seevers 1951, Klimaszewski et al. 2009b, 2011). The adults were collected in August and September.

##### Gyrophaena uteana

Taxon classificationAnimaliaColeopteraStaphylinidae

Casey

Gyrophaena uteana (for diagnosis and illustrations, see Seevers 1951, Brunke et al. 2012, Webster et al. 2012)

###### Distribution.

**Table:** Distribution of *Gyrophaena uteana*

Origin	Nearctic
Distribution	Canada: AB, BC, NB, ON, QC, **SK**; USA: CA, CO, UT
New records	New provincial records: **Saskatchewan:** Prince Albert, aspen stand, 54.7217°, -105.6896°, 484 m, 5.VI.2013(LFC)1 male; La Ronge, alder/spruce litter, 55.118°, -105.2457°, 366 m, 6.VI.2013(BGC)1 male
References	Casey 1906, Seevers 1951, genitalia in Klimaszewski et al. 2009b (as *Gyrophaena gaudens*), Brunke et al. 2012, Webster et al. 2012, Bousquet et al. 2013

###### Natural history.

The Saskatchewan specimens were found in June in an aspen stand and in alder/spruce litter in a forest.

##### Homalota plana

Taxon classificationAnimaliaColeopteraStaphylinidae

(Gyllenhal)

Homalota plana (for diagnosis and illustrations, see Klimaszewski et al. 2011)

###### Distribution.

**Table:** Distribution of *Homalota plana*

Origin	Palaearctic; adventive in North America
Distribution	Canada: **AB**, NB, NF, NS; USA: AK; Palaearctic: Europe, North Africa, Asia
New records	New provincial records: **Alberta:** c. 100 km NW of Peace River, Blk C31, 10-year-old regenerating coniferous stands, 56.6833°, -118.6336°, 8.IX.2011 (NoFC) 1 female; Blk C31, intact white spruce forest, white spruce girdled in 2010, 56.7114°, -118.6470°, 20.VI.2010 (NoFC) 1 male and 1 female; EMEND compartment 892, white spruce snag, 56.7506°, -118.4001°, 781.1 m, 15.IX.2009 (NoFC) 1 female; EMEND compartment 896, white spruce girdled in 2009, 56.7572°, -118.3962°, 802.7 m, 6.IX.2010 (NoFC) 1 female; EMEND compartment 898, white spruce girdled in 2009, 56.7598°, -118.3990°, 826.3 m, 13.VIII.2010 (NoFC) 1 female
References	Gouix and Klimaszewski 2007, Klimaszewski et al. 2011, Bousquet et al. 2013

###### Natural history.

In Alberta, most adults were collected using window traps attached to boles of recently girdled trees and snags of white spruce in white spruce dominated stands. Elsewhere, adults were found under bark of coniferous trees (Klimaszewski et al. 2007a, 2011). The adults were collected from June to September.

#### MYLLAENINI Ganglbauer

##### Myllaena arcana

Taxon classificationAnimaliaColeopteraStaphylinidae

Casey

Myllaena arcana (for diagnosis and illustrations, see Klimaszewski et al. 2011)

###### Distribution.

**Table:** Distribution of *Myllaena arcana*

Origin	Nearctic
Distribution	Canada: AB, LB, NB, NF, NS, ON, QC, **SK**; USA: AL, FL, IA, IL, MA, NH, NJ; Mexico
New records	New provincial record: **Saskatchewan:** Meadow Lake, birch/alder litter, 54.4188°, -108.944°, 482 m, 7.VI.2013(BGC)1 female
References	Klimaszewski 1982, Campbell and Davies 1991, Gouix and Klimaszewski 2007, Bishop et al. 2009, Majka and Klimaszewski 2010, Klimaszewski et al. 2008, 2011, Bousquet et al. 2013

###### Natural history.

In Saskatchewan, adults were captured in birch and alder litter in a forest. Elsewhere, the species appears to be riparian. Adults were collected from February to November from debris near streams and lakes, and from a beaver lodge (Klimaszewski et al. 2008, 2011).

#### OXYPODINI C.G. Thomson

##### Devia prospera

Taxon classificationAnimaliaColeopteraStaphylinidae

(Erichson)

Devia prospera (for diagnosis and illustrations, see Klimaszewski et al. 2011)

###### Distribution.

**Table:** Distribution of *Devia prospera*

Origin	Holarctic
Distribution	Canada: AB, BC, LB, MB, NB, NT, ON, **SK**; USA: AK, CO, MI, MN, NM, OR, SD, UT, WA, WY; Palaearctic: Europe, Asia
New records	New provincial records: **Saskatchewan:** La Ronge, alder/spruce litter, 55.118°, -105.2457°, 366 m, 6.VI.2013(BGC)1 male; Meadow Lake, wet spruce litter, 54.4144°, -108.8897°, 486 m, 7.VI.2013 (BGC, LFC) 1 male, 2 females
References	Gusarov 2003a, Gouix and Klimaszewski 2007, Klimaszewski et al. 2007a, 2011, Webster et al. 2009, Majka and Klimaszewski 2010, Bousquet et al. 2013

###### Natural history.

In Saskatchewan, adults were collected from alder/spruce and wet spruce litter in forests. In Newfoundland, adults were collected in abundance using pitfall traps in a patch of mixedwood forest in an urban area but were uncommon in a disturbed field with forbs and grasses (Klimaszewski et al. 2011). Elsewhere, adults were collected in human settlements from stables, barns, heaps of straw, haystacks, rotting organic debris, mushrooms, and forest litter (Klimaszewski et al. 2007a). The adults were collected from June to August.

##### Ocyusa canadensis

Taxon classificationAnimaliaColeopteraStaphylinidae

Lohse

Ocyusa canadensis (for diagnosis and illustrations, see Lohse et al. 1990, Klimaszewski et al. 2014)

###### Distribution.

**Table:** Distribution of *Ocyusa canadensis*

Origin	Nearctic
Distribution	Canada: NB, NF, ON, **SK**, YT; USA: AK
New records	New provincial record: **Saskatchewan:** Cypress Hills, wet willow stand, 49.5978°, -109.9231°, 1134 m, 2.IX.2012 (BGC, LFC) 3 males, 2 females
References	Lohse et al. 1990, Klimaszewski et al. 2014

###### Natural history.

The Saskatchewan specimens were captured in a wet willow stand. Elsewhere, adults were collected at lake margins, on moist soil/gravel among sedges and by treading *Carex* and grasses (Klimaszewski et al. 2014). The adults were collected from June to September.

##### Oxypoda grandipennis

Taxon classificationAnimaliaColeopteraStaphylinidae

(Casey)

Oxypoda grandipennis (for diagnosis and illustrations, see Klimaszewski et al. 2006, 2011)

###### Distribution.

**Table:** Distribution of *Oxypoda grandipennis*

Origin	Nearctic
Distribution	Canada: AB, BC, LB, NB, NF, NS, ON, QC, **SK**, YT; USA: AK, NH
New records	New provincial record: **Saskatchewan:** Cypress Hills, 49.669°, -109.4998°, 1196 m, 2.IX.2012(BGC)1 male
References	Klimaszewski et al. 2005, 2006, 2011, Gouix and Klimaszewski 2007, Majka and Klimaszewski 2010, Bousquet et al. 2013

###### Natural history.

In Newfoundland, adults were collected using pitfall traps, carrion traps and flight intercept traps in various forest types (coniferous, deciduous, mixedwood and riparian) and on coastal limestone barrens of Labrador (Klimaszewski et al. 2011). Some specimens were collected from rotting mushrooms in forests (Klimaszewski et al. 2011). Elsewhere, adults were collected from leaf litter, moss, fungi, in natural and harvested deciduous and coniferous forests (Klimaszewski et al. 2006). It is a very adaptable and common *Oxypoda* species in Canada. The adults were collected from May to October.

##### Oxypoda hiemalis

Taxon classificationAnimaliaColeopteraStaphylinidae

Casey

Oxypoda hiemalis (for diagnosis and illustrations, see Klimaszewski et al. 2011)

###### Distribution.

**Table:** Distribution of *Oxypoda hiemalis*

Origin	Nearctic
Distribution	Canada: **AB**, LB, NB, NF, NS, NT, ON QC; USA: AK
New records	New provincial record: **Alberta:** c. 90 km NW of Peace River, EMEND compartment 918, white spruce girdled in 2009, 56.7923°, -118.3634°, 7.VIII.2009 (NoFC) 1 female
References	Klimaszewski et al. 2011, Bousquet et al. 2013

###### Natural history.

In Alberta, a single female was collected in a white spruce dominated stand using a window trap installed on the trunk of a recently girdled white spruce. Elsewhere, adults were captured in various forest types, agricultural fields, a disturbed meadow with *Salix* shrubs, and vegetation on coastal sand dunes (Klimaszewski et al. 2011). The adults were collected from March to October.

##### Oxypoda lacustris

Taxon classificationAnimaliaColeopteraStaphylinidae

Casey

Oxypoda lacustris (for diagnosis and illustrations, see Klimaszewski et al. 2006, 2011)

###### Distribution.

**Table:** Distribution of *Oxypoda lacustris*

Origin	Nearctic
Distribution	Canada: AB, BC, LB, **MB**, NB, NF, NS, NT, QC, ON, **SK**, YT; USA: AK
New records	New provincial records: **Saskatchewan:** Lug Creek, spruce/alder litter, 55.1776°, -106.6885°, 406 m, 6.VI.2013(BGC)1 female; La Ronge, alder/spruce litter, 55.118°, -105.2457°, 366 m, 6.VI.2013(BGC)1 female; Prince Albert, sandy beach, 53.9804°, -106.28°, 532 m, (LFC) 1 male; **Manitoba:** Winnipeg, Whittier Park, river bank litter, 49.8996, -97.1250, 228 m, 18.X.2009(BGC)4 males, 6 females
References	Klimaszewski et al. 2005, 2006, Gouix and Klimaszewski 2007, Webster et al. 2009, Majka and Klimaszewski 2010

###### Natural history.

In Saskatchewan and Manitoba, specimens were found in alder/spruce litter in a forest stand and in litter on river banks. In Newfoundland, adults were collected using pitfall traps in birch forests, burned forest, fir forest, coastal sand dunes and coastal barrens (Klimaszewski et al. 2011). Elsewhere, adults were collected in forest litter, moss, gopher burrows, and muskrat nests (Klimaszewski et al. 2006, Webster et al. 2009). The adults were collected from June to September.

##### Oxypoda orbicollis

Taxon classificationAnimaliaColeopteraStaphylinidae

Casey

Oxypoda orbicollis (for diagnosis and illustrations, see Klimaszewski et al. 2006, 2011)

###### Distribution.

**Table:** Distribution of *Oxypoda orbicollis*

Origin	Nearctic
Distribution	Canada: AB, NF, NS, ON, QC, **SK**, YT; USA: WI
New records	New provincial record: **Saskatchewan:** Cypress Hills, mushroom, pine forest, 49.669°, -109.4998°, 1196 m, 2.IX.2012 (BGC, LFC) 2 males
References	Klimaszewski et al. 2005, 2006, 2011, Gouix and Klimaszewski 2007, Majka and Klimaszewski 2010, Bousquet et al. 2013

###### Natural history.

In Saskatchewan, specimens were found on a mushroom in a pine forest. In Labrador, specimens were collected using pitfall traps in various coniferous, deciduous and mixedwood forest types (Klimaszewski et al. 2011). Elsewhere, adults were collected in forest litter in deciduous-dominated stands and in balsam fir forest, as well as in sphagnum moss (Klimaszewski et al. 2006). The adults were collected from June to September.

##### Oxypoda pseudolacustris

Taxon classificationAnimaliaColeopteraStaphylinidae

Klimaszewski

Oxypoda pseudolacustris (for diagnosis and illustrations, see Klimaszewski et al. 2006, 2011)

###### Distribution.

**Table:** Distribution of *Oxypoda pseudolacustris*

Origin	Nearctic
Distribution	Canada: AB, NB, NF, NS, ON, QC, **SK**
New records	New provincial record: **Saskatchewan:** Prince Albert, aspen stand, 54.7217°, -105.6896°, 484 m, 5.VI.2013(BGC)1 female.
References	Klimaszewski et al. 2005, 2006, 2011, Gouix and Klimaszewski 2007, Majka and Klimaszewski 2010, Bousquet et al. 2013

###### Natural history.

In Saskatchewan, adults were captured in an aspen stand. In Newfoundland, adults were reared from the boles of dead balsam fir, collected with pitfall traps in fir forests and collected from coastal sand dunes (Klimaszewski et al. 2011). Elsewhere, adults were collected mostly from sphagnum moss near small bodies of water, other moss, forest litter in coniferous and deciduous forests and organic litter in alpine and subalpine habitats. Most adults were collected from May through October, with some records from November and December (details in Klimaszewski et al. 2006).

#### PLACUSINI Mulsant & Rey

##### Placusa incompleta

Taxon classificationAnimaliaColeopteraStaphylinidae

Sjöberg

Placusa incompleta (for diagnosis and illustrations, see Klimaszewski et al. 2001, 2011)

###### Distribution.

**Table:** Distribution of *Placusa incompleta*

Origin	Palaearctic, adventive in North America; possibly introduced separately in eastern Canada and western WA
Distribution	Canada: **AB**, BC, NB, NF, NS, QC; USA: WA; Palaearctic: Europe
New records	New provincial records: **Alberta:** c. 100 km NW of Peace River, Blk C14, white spruce gridled in 2010, 56.707°, -118.778°, 24.VIII.2011 (NoFC) 1 female; EMEND compartment 898, white spruce log in early decay stage, 56.759°, -118.399°, 826.3 m, 10.VII.2010 (NoFC) 2 females; EMEND compartment 889, white spruce snag, 56.7498°, -118.4188°, 27.VII.2010 (NoFC) 1 female; EMEND compartment 892, white spruce snag, 56.7506°, -118.4001°, 781.1 m, 10.VII.2010 (NoFC) 1 female; EMEND compartment 919, white spruce snag, 56.7954°, -118.3610°, 714.3 m, 18.VI.2010 (NoFC) 1 female; EMEND compartment 920, white spruce snag, 56.7906°, -118.3740°, 10.VII.2010 (NoFC) 1 female; EMEND compartment 920, white spruce gridled in 2009, 56.7921°, -118.3737°, 17.IX.2009 (NoFC) 1 female
References	Klimaszewski et al. 2001, 2011, Bousquet et al. 2013

###### Natural history.

In Alberta, adults were collected from dead or dying white spruce in aggregated retention patches surrounded by different levels of dispersed retention, using emergence traps and window traps. Elsewhere, adults were found in various deciduous and coniferous forests, using a pit-light trap and ethanol-baited Lindgren funnel traps (Klimaszewski et al. 2001, 2011). The adults in northwestern Alberta were collected from June to September.

##### Placusa pseudosuecica

Taxon classificationAnimaliaColeopteraStaphylinidae

Klimaszewski

Placusa pseudosuecica (for diagnosis and illustrations, see Klimaszewski et al. 2001)

###### Distribution.

**Table:** Distribution of *Placusa pseudosuecica*

Origin	Nearctic
Distribution	Canada: **AB**, BC, QC, ON
New records	New provincial records: **Alberta:** c. 100 km NW of Peace River, Blk C31, white spruce snag, 56.697°, -118.652°, 13.VII.2010 (NoFC) 1 female; Blk C14, white spruce gridled in 2010, 56.686°, -118.643°, 5.VIII.2011 (NoFC) 1 female; Blk C14, white spruce girdled in 2010, 56.712°, -118.779°, 16.VIII.2010 (NoFC) 1 female; Blk 79A, white spruce girdled in 2010, 56.688°, -118.605°, 14.VII.2010 (NoFC) 1 female; EMEND compartment 920, white spruce girdled in 2009, 56.7908°, -118.3738°, 18.VII.2009 (NoFC) 1 female; EMEND compartment 929, white spruce girdled in 2009, 56.8024°, -118.3226°, 29.VII.2010 (NoFC) 1 female; EMEND compartment 933, white spruce girdled in 2009, 56.8058°, -118.3324°, 17.VII.2009 (NoFC) 1 female
References	Klimaszewski et al. 2001, Bousquet et al. 2013

###### Natural history.

In Alberta, adults were collected from dead or dying white spruce in aggregated retention patches surrounded by different levels of dispersed retention, using window traps. Elsewhere, adults were found in mature coniferous forests, using pit-light traps and ethanol-baited Lindgren funnel traps (Klimaszewski et al. 2011). The adults were collected in July and August.

##### Placusa tachyporoides

Taxon classificationAnimaliaColeopteraStaphylinidae

(Waltl)

Placusa tachyporoides (for diagnosis and illustrations, see Klimaszewski et al. 2001)

###### Distribution.

**Table:** Distribution of *Placusa tachyporoides*

Origin	Palaearctic, adventive in North America
Distribution	Canada: **AB**, BC, NB, NS, QC, ON; Palaearctic: Europe, the Mediterranean, Caucasus, Siberia, Japan
New records	New provincial record: **Alberta:** c. 90 km NW of Peace River, EMEND compartment 918, white spruce logs, 56.792°, -118.364°, 757.8 m, 14.VI.2010 (NoFC) 2 males and 1 female in early decay stage and 1 female in intermediate decay stage
References	Klimaszewski et al. 2001, Bousquet et al. 2013

###### Natural history.

In Alberta, adults were reared from white spruce logs in early and intermediate decay stages in white spruce dominated stands. Elsewhere, adults were found in various deciduous and coniferous forests, using a flight intercept trap, ethanol-baited Lindgren funnel traps, pit-light traps, and pitfall traps.

## Supplementary Material

XML Treatment for Aleochara (s. str.) sekanai

XML Treatment for Tinotus morion

XML Treatment for Acrotona recondita

XML Treatment for Aloconota sulcifrons

XML Treatment for Atheta (Datomicra) celata

XML Treatment for Atheta (Datomicra) dadopora

XML Treatment for Atheta (Datomicra) nigra

XML Treatment for Atheta (Bessobia) cryptica

XML Treatment for Atheta (Dimetrota) fanatica

XML Treatment for Atheta (Atheta) graminicola

XML Treatment for Atheta (Pseudota) klagesi

XML Treatment for Atheta (Chaetida) longicornis

XML Treatment for Atheta (Microdota) platonoffi

XML Treatment for Atheta (Dimetrota) prudhoensis

XML Treatment for Atheta (Microdota) pseudosubtilis

XML Treatment for Atheta (Alaobia) ventricosa

XML Treatment for Boreophilia davidgei

XML Treatment for Boreophilia islandica

XML Treatment for Boreostiba parvipennis

XML Treatment for Dinaraea pacei

XML Treatment for Dinaraea worki

XML Treatment for Liogluta aloconotoides

XML Treatment for Lypoglossa franclemonti

XML Treatment for Philhygra botanicarum

XML Treatment for Philhygra clemens

XML Treatment for Philhygra jarmilae

XML Treatment for Philhygra ripicoloides

XML Treatment for Philhygra rostrifera

XML Treatment for Philhygra sinuipennis

XML Treatment for Philhygra terrestris

XML Treatment for Schistoglossa campbelli

XML Treatment for Schistoglossa carexiana

XML Treatment for Schistoglossa hampshirensis

XML Treatment for Seeversiella globicollis

XML Treatment for Trichiusa pilosa

XML Treatment for Gymnusa campbelli

XML Treatment for Gyrophaena criddlei

XML Treatment for Gyrophaena insolens

XML Treatment for Gyrophaena uteana

XML Treatment for Homalota plana

XML Treatment for Myllaena arcana

XML Treatment for Devia prospera

XML Treatment for Ocyusa canadensis

XML Treatment for Oxypoda grandipennis

XML Treatment for Oxypoda hiemalis

XML Treatment for Oxypoda lacustris

XML Treatment for Oxypoda orbicollis

XML Treatment for Oxypoda pseudolacustris

XML Treatment for Placusa incompleta

XML Treatment for Placusa pseudosuecica

XML Treatment for Placusa tachyporoides
